# Synthesis and Bio-Evaluation of Natural Butenolides-Acrylate Conjugates

**DOI:** 10.3390/molecules24071304

**Published:** 2019-04-03

**Authors:** Longzhu Bao, Shuangshuang Wang, Di Song, Jingjing Wang, Xiufang Cao, Shaoyong Ke

**Affiliations:** 1 College of Science, Huazhong Agricultural University, Wuhan 430070, China; baolz@webmail.hzau.edu.cn (L.B.); 1140291490@webmail.hzau.edu.cn (S.W.); songdi@webmail.hzau.edu.cn (D.S.); 13523041961@163.com (J.W.); 2 National Biopesticide Engineering Research Center, Hubei Biopesticide Engineering Research Center, Hubei Academy of Agricultural Science, Wuhan 430064, China

**Keywords:** 3-aryl-4-hydroxy-2(5*H*)-furanone, butenolides, acrylate, antifungal activity, natural product

## Abstract

A series of novel 3-aryl-4-hydroxy-2(5*H*) furanone-acrylate hybrids were designed and synthesized based on the natural butenolides and acrylates scaffolds. The structures of the prepared compounds were characterized by ^1^H-NMR, ^13^C-NMR and electrospray ionization mass spectrometry (ESI-MS), and the bioactivity of the target compounds against twelve phytopathogenic fungi was investigated. The preliminary in vitro antifungal activity screening showed that most of the target compounds had moderate inhibition on various pathogenic fungi at the concentration of 100 mg·L^−1^, and presented broad-spectrum antifungal activities. Further studies also indicated that compounds **7e** and **7k** still showed some inhibitory activity against *Pestallozzia theae*, *Sclerotinia sclerotiorum* and *Gibberella zeae* on rape plants at lower concentrations, which could be optimized as a secondary lead for further research.

## 1. Introduction

Natural products play an important role in the field of pesticides, and structural modification and transformation using natural products as a precursor are important ways to create new candidate pesticides. It has the advantages of a high target, high efficiency and low toxicity [1]. As a typical example, synthetic strobilurins are now an important class of fungicides for crop protection, they were derived from structural optimization by taking the sterilizing active natural antibiotic Strobilurin A as a precursor. During the period of 2011–2017, up to five Strobilurin fungicides have been registered, and which are Coumoxystrobin, Fluoxastrobin, Mandestrobin, Metominostrobin, Picoxystrobin [2,3]. This class of fungicides has good activity for almost all fungal diseases, such as powdery mildew, rust, blight, net blotch, downy mildew, rice blast, etc, and can not only effectively prevent and treat resistance to other fungicides but also has the effect of prolonging the vegetative growth of crops and increasing yields [4]. More and more researchers are involved in the development of this kind of fungicides.

**Figure 1:**
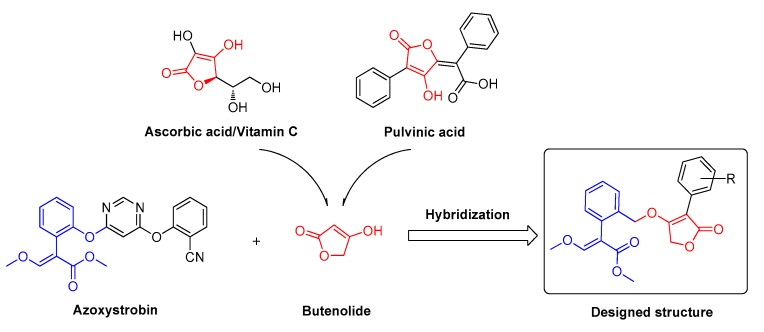
Design strategy of natural butenolides-acrylate conjugates.

The structure of traditional strobilurin fungicide includes pharmacophore, aromatic bridge and side chain. Many studies have shown that the modification of the aromatic bridge of this fungicide does not significantly affect their biological activity, therefore the structural modification is mainly focused on the side chain. The core structure of this fungicide, the acrylate structure unit, often remains unchanged [5,6]. 3-Aryl-4-hydroxy-2(5*H*)-furanone belongs to the class of natural butenolides, and the core structure of these compounds is a five-membered lactone ring unit, and which is not only a structural fragment contained in many natural products [7,8] but also the core structure of insecticides [9]. As shown in Figure 1, many natural products such as ascorbic acid (vitamin C) and pulvinic acid possess the β-keto-γ-butyrolactone motif, and a lot of butenolide compounds containing this moiety have anti-tumor, anti-inflammatory, antioxidant and other biological activities [10,11,12,13,14,15,16,17]. Studies have shown that in the process of bacterial cell wall synthesis, these butenolide derivatives can inhibit the activity of Mur enzyme to achieve antibacterial action, so it is an antibacterial lead compound with research and development potential [18,19].

In this paper, to develop compounds with better antifungal activity, we combined different substituted butenolide structural units with the acrylate structural unit to obtain a series of 3-aryl-4-hydroxy-2(5*H*)-furanone-acrylate hybrids, which was shown in Figure 1. In addition, the target compounds were tested for their antifungal activity against twelve pathogenic fungi, and the results showed that the target compounds had moderate antifungal activities.

## 2. Results and Discussion

### 2.1. Synthesis of Target Compounds

**Scheme 1:**
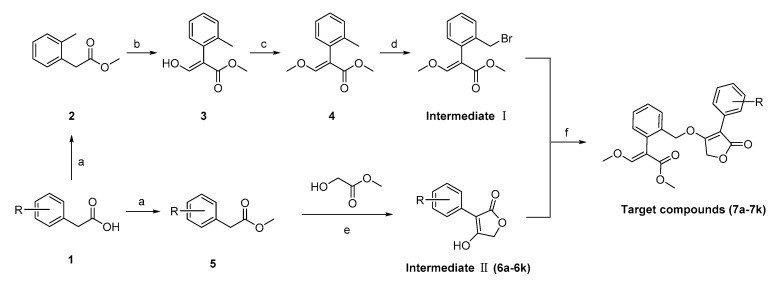
Synthetic route of the target compounds. **7a**: R = 2-Me, **7b**: R = 2,4-Cl_2_, **7c**: R = 4-F, **7d**: R = H, **7e**: R = 2-Cl, **7f**: R = 4-Cl, **7g**: R = 2,6-Cl_2_, **7h**: R = 2-MeO, **7i**: R = 4-Me, **7j**: R = 2-F, **7k**: R = 2,4,5-F_3_. Reagents and conditions: (a) CH_3_OH, concentrated sulfuric acid, reflux; (b) NaH, HCOOCH_3_, toluene; (c) K_2_CO_3_, (CH_3_)_2_SO_4_, *N*,*N*-dimethylformamide (DMF); (d) CCl_4_, *N*-bromosuccinimide (NBS), azodiisobutyronitrile (AIBN); (**e**) *t*-BuOK, THF, reflux; (**f**) K_2_CO_3_, CH_3_CN, room temperature.

The synthetic route for target compounds is shown in Scheme 1. Firstly, 2-(*O*-tolyl)acetic acid was used as a raw material to obtain key intermediates I by esterification, aldol condensation, methylation and bromination, that is methyl-2-(2-(bromomethyl)phenyl)-3-methoxyacrylate. Next, the key intermediate II (**6a**–**6k**) was obtained by Dickman condensation reaction using substituted methyl 2-phenylacetate **5** and methyl 2-hydroxyacetate as substances. Finally, intermediate I is reacted with intermediates II (**6a**–**6k**) to obtain the target compounds **7a**–**7k**.

### 2.2. Speculated Synthetic Mechanism of the Key Intermediate II

**Scheme 2:**
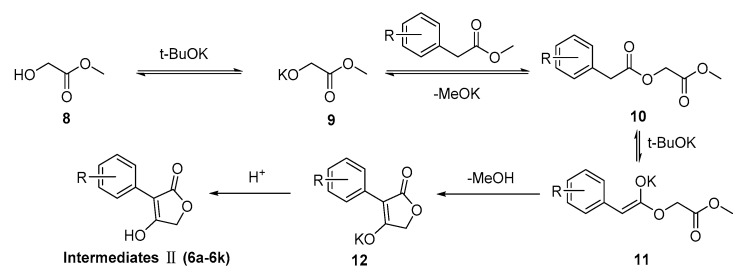
Speculated synthetic mechanism of the key intermediates II (**6a**–**6k**).

Based on the above experimental results, the possible mechanism for the formation of the key intermediates II is presumed as follows (Scheme 2): it mainly involves a transesterification and a Dieckmann condensation as the two main steps. The transesterification of methyl 2-phenylacetate **5** (with different substituents on the phenyl ring) and methyl 2-hydroxyacetate **8** by the alkoxide **9** derived from methyl glycolate **8** to obtain ester **10**, which is then converted to the key intermediates II by the usual Dieckmann condensation.

### 2.3. Spectral Analysis of Target Compounds

The target compounds were identified by ^1^H-NMR, ^13^C-NMR and ESI-MS, the results of these spectra indicate that the structure of these compounds is correct (Supplementary Material). The compound **7g** was used as an example for analysis. In the ^1^H-NMR spectra of this compound, the chemical shift value of the hydrogen on the methylene group in benzylic position and the hydrogen on the methylene group on the saturated carbon atom in the five-membered ring is similar, 4.76 and 4.85 respectively. The δ values of hydrogens on the benzene ring are in region 7.00–7.50 ppm, and the signal multiplicity, difficult to be assigned, has been indicated as multiplet. In the ^13^C-NMR spectra of this compound, the chemical shifts of the carbon of the methoxy groups of the methoxy acrylate skeleton appear in the high field region, at 62.15 and 51.83 ppm, while the 13C signals of the carbonyl groups mainly appear in the low field region, at 173.51 and 171.45 ppm. In addition, the mass spectra also showed molecular ion peaks at the appropriate *m*/*z* values, which further confirmed the structure of target compounds.

### 2.4. Biological Activity

#### 2.4.1. Primary Fungicidal Activities

The in vitro fungicidal activities of all compounds and some intermediates against 12 different phytopathogenic fungi at a concentration of 100 mg·L^−1^ are listed in Table 1. The results of preliminary bioassays were compared with those of Azoxystrobin, Carbendazim, intermediates I and II.

As indicated in Table 1, under dosage condition of 100 mg·L^−1^, most of the compounds showed potent fungicidal activities against 12 different phytopathogenic fungi, especially for the *Phomopsis adianticola* and *Rap Sclerotinia stem rot.* Compound **7e** has the most obvious inhibitory effect on all fungi, and the inhibition effect on *Rhizoctonia solani*, *Phomopsis adianticola*, *Rap Sclerotinia stem rot*, *Pestallozzia theae* and *Monilinia fructicola* is better than on those of other fungi, the inhibition rate against *Rap Sclerotinia stem rot* is up to 72.99% that better than the intermediate I. Compound **7g** has a good selectivity for the inhibition of *Phomopsis adianticola.* Both compounds **7a** and **7k** have more prominent inhibition on the *Phomopsis adianticola* and *Rap Sclerotinia stem rot.* Compounds **7b**, **7c**, **7d** perform well on the inhibition against *Rap Sclerotinia stem rot*.

From the data in Table 1, we can also see that compared with the intermediate II (**6c**), the antifungal activity for most of the fungi except for *Pestalotiopsis theae* of the target compound **7c** is significantly improved after hybridization. Compared with the intermediate I, the antifungal activity of the compounds after hybridization has not been improved, which also indicates that the antifungal activity of these compounds may depend mainly on the methoxy acrylate skeleton structure.

#### 2.4.2. Further Fungicidal Activities

Based on the above antifungal activity, we launched a series of screening test work in a lower concentration range.

The inhibitory effect of compounds **7k** and **7e** on four common plant pathogenic fungi can be seen in Table 2. From the table, we can see that as the concentration gradient decreases, the inhibition rate of compounds **7k** and **7e** against most fungi is generally reduced, which is consistent with our expected results. What surprised us was that under the same concentration conditions, the inhibitory effect of them on *Gibberella zeae* and *Sclerotinia sclerotiorum* fungi strains are significantly better than the control of intermediate I. For other strains, these two compounds did not show good inhibitory activity, indicating that the compounds have good selectivity for *Gibberella zeae* and *Sclerotinia sclerotiorum* fungi strains.

### 2.5. Analysis of Structure-Activity Relationships

From the perspective of compound structure, this paper mainly discusses the effect of different substituents on the benzene ring on the antifungal activity of the target compounds. As can be seen from Table 1, when the substituent on the benzene ring is an electron-donating group, such as the compound **7i**, the antifungal activity of the compound is adversely affected, and the electron-donating group weakens the activity of the compound. Conversely, when a substituent is an electron-withdrawing group, such as compound **7c**, the activity of the compound is much higher than that of the intermediates. Therefore, the antifungal action of the novel synthesized 3-aryl-4-hydroxy-2(5*H*) furanone-acrylate hybrid strobilurin derivatives is mainly caused by the methoxy acrylate structural unit, and the introduction of this main structural unit can enhance the antifungal action of the intermediate II.

## 3. Conclusions

A series of novel 3-aryl-4-hydroxy-2(5*H*) furanone-acrylate hybrids containing a five-membered lactone structure were designed and synthesized based on the structural characteristics of methoxyacrylate backbone. The test for fungicidal activity indicated that these compounds have a moderate inhibitory effect on most plant pathogenic fungi, and showing good selectivity for *Rap Sclerotinia stem rot* fungi strain, which could be further optimized as a secondary lead.

## 4. Experimental Section

### 4.1. Instruments and Reagents

All chemicals or reagents used for syntheses were commercially available, were of AR grade, and were used as received without further purification except as indicated. Anhydrous CH_2_Cl_2_ and CH_3_CN were dried according to standard methods. All other solvents and reagents were analytical reagent and used directly without purification. All melting points (m. p) were obtained with a digital model X-4 apparatus (Shanghai Instrument Physical Optics Instrument Co., LTD, Shanghai, China) and are uncorrected. ^1^H-NMR spectra were recorded on a Brucker spectrometer (Bruker, Bremen, Germany) at 600 MHz with CDCl_3_ as the solvent and TMS as the internal standard; ^13^C-NMR spectra were recorded on a Brucker spectrometer at 150 MHz with CDCl_3_ as the solvent. Chemical shifts are reported in δ (parts per million) values. Coupling constants ^n^*J* are reported in Hz. Liquid chromatography–mass spectrometry analysis was performed on a Waters ACQUITY UPLC^®^ H-CLASS PDA (Waters^®^, Milford, MA, USA) instrument. Analytical thin-layer chromatography (TLC) was carried out on precoated plates, and spots were visualized with ultraviolet light.

### 4.2. Experimental Method

#### 4.2.1. Synthesis of Methoxyacrylate Intermediates

Methyl-2-(2-(bromomethyl)phenyl)-3-methoxyacrylate was prepared according to known methods as shown in Scheme 1 [20,21,22].

#### 4.2.2. General Procedure for Synthesis of the Key Lactone Intermediate II (**6a**–**6k**)

General synthetic procedure for the key lactone intermediates II (**6a**–**6k**), for example **6a**. 2-(*O*-tolyl) acetic acid (0.15 g, 1 mmol) was dissolved in 15 mL of methanol, slowly add 2–3 drops of concentrated sulfuric acid, then refluxing for 8 h and monitored by TLC, after the reaction is completed, the solvent methanol was removed by rotary evaporation, 30 mL of water was added to the residue and stirred, extracted three times with ethyl acetate, washed with water, dried and concentrated to give compound **5a**. The obtained compound **5a** was placed in a round bottom flask, and 1.2 eq of methyl glycolate, 20 mL of tetrahydrofuran, and 2.2 eq of potassium t-butoxide were added, refluxing and stirring the reaction and monitored by TLC, after the reaction is completed, 50 mL of water was added to the residue and stirred, adjust the pH of the solution to 5–6, then extracted three times with ethyl acetate, washed with water, dried and concentrated to obtain key lactone intermediates II (**6a**) [10,11,12,13,14].

#### 4.2.3. General Procedure for Synthesis of Target Compounds (**7a**–**k**)

General synthetic procedure for the target compounds, take **7a** for example. 4-Hydroxy-3-(*O*-tolyl)furan-2(5*H*)-one (0.19 g, 1 mmol) was dissolved in 10 mL of acetonitrile, and anhydrous potassium carbonate (0.21 g, 1.5 mmol) was added to the solution in a round bottom flask. The solution was stirred for 0.5 h, and methyl-2-(2-(bromomethyl)phenyl)-3-methoxyacrylate (0.29 g, 1 mmol) was then added. The reaction is carried out at room temperature and monitored by TLC. After 10 h, the mixture was diluted with 50 mL water and extracted with ethyl acetate (3 × 60 mL). The combined extracts were washed with brine, dried (anhydrous sodium sulfate) and filtered. The filtrate was evaporated, and the crude product was purified via silica gel column chromatography using a 1:5 (*v*/*v*) mixture of ethyl acetate and petroleum ether (boiling point range 60–90 °C) as the eluting solution to obtain the pure target compound **7a**. The specific experimental result data is as follows:

*Data for Methyl 3-methoxy-2-(2-(((5-oxo-4-(O-tolyl)-2,5-dihydrofuran-3-yl)oxy)methyl)phenyl)acrylate* (**7a**). Yield: 37.1%; light yellow oil; ^1^H-NMR (600 MHz, chloroform-*d*) δ 7.41 (s, 1H, CH_3_OCH), 7.34–7.30 (m, 3H, ArH), 7.27 (d, *J* = 7.2 Hz, 1H, ArH), 7.26–7.18 (m, 3H, ArH), 7.15–7.11 (m, 1H, ArH), 4.80 (s, 2H, ArCH**_2_**), 4.70 (s, 2H, CH_2_), 3.67 (s, 3H, CH**_3_**OCH), 3.64 (s, 3H, CH**_3_**OCO), 2.26 (s, 3H, ArCH**_3_**). ^13^C-NMR (150 MHz, chloroform-*d*) δ 173.32, 172.01, 167.38, 160.29, 138.12, 133.89, 131.64, 131.33, 131.11, 130.24, 129.32, 128.79, 128.64, 128.29, 127.69, 125.74, 109.46, 104.09, 72.00, 66.83, 62.12, 51.82, 20.26. MS (ESI) *m*/*z* 417.38 [M + Na]^+^, calcd. For C_23_H_22_O_6_ *m*/*z* = 394.14.

*Data for Methyl 2-(2-(((4-(2,4-dichlorophenyl)-5-oxo-2,5-dihydrofuran-3-yl)oxy)methyl)phenyl)-3-methoxyacrylate* (**7b**). Yield: 31.2%; yellow oil; ^1^H-NMR (600 MHz, chloroform-*d*) δ 7.49 (d, *J* = 1.9 Hz, 1H, CH_3_OCH), 7.47 (s, 1H, ArH), 7.35 (s, 3H, ArH), 7.30 (d, *J* = 1.9 Hz, 1H, ArH), 7.30 (s, 1H, ArH), 7.15 (d, *J* = 2.7 Hz, 1H, ArH), 4.89 (s, 2H, ArCH**_2_**), 4.71 (s, 2H, CH**_2_**), 3.74 (s, 3H, CH**_3_**OCH), 3.66–3.66 (s, 3H, CH**_3_**OCO). ^13^C-NMR (150 MHz, chloroform-*d*) δ 173.47, 172.39, 167.35, 160.33, 135.86, 135.39, 133.48, 133.30, 131.62, 131.44, 129.55, 128.82, 128.40, 127.89, 127.73, 127.26, 109.48, 101.17, 72.00, 66.91, 62.20, 51.87. MS (ESI) *m*/*z* 471.31 [M + Na]^+^, calcd. For C_22_H_18_Cl_2_O_6_ *m*/*z* = 448.05.

*Data for Methyl 2-(2-(((4-(4-fluorophenyl)-5-oxo-2,5-dihydrofuran-3-yl)oxy)methyl)phenyl)-3-methoxyacrylate* (**7c**). Yield: 35.4%; yellow oil; ^1^H-NMR (600 MHz, chloroform-*d*) δ 7.95 (dd, *J* = 8.9, 5.5 Hz, 2H, ArH), 7.57 (s, 1H, CH_3_OCH), 7.45–7.35 (m, 3H, ArH), 7.23–7.17 (m, 1H, ArH), 7.08 (t, *J* = 8.8 Hz, 2H, ArH), 5.12 (s, 2H, ArCH**_2_**), 4.70 (s, 2H, CH_2_), 3.76 (s, 3H, CH_3_OCH), 3.67 (s, 3H, CH**_3_**OCO). ^13^C-NMR (150 MHz, chloroform-*d*) δ 172.54, 172.26, 167.44, 162.01 (d, ^1^*J_C-F_* = 246 Hz), 160.62, 133.80, 131.86, 131.69, 129.47 (d, ^3^*J_C-F_* = 7.5 Hz), 129.00, 128.68, 127.49, 125.58 (d, ^4^*J_C-F_* = 3 Hz), 115.32 (d, ^2^*J_C-F_* = 18 Hz), 109.66, 102.26, 71.36, 64.89, 62.31, 51.96. MS (ESI) *m*/*z* 421.35 [M + Na]^+^, calcd. For C_22_H_19_FO_6_ *m*/*z* = 398.12.

*Data for Methyl 3-methoxy-2-(2-(((5-oxo-4-phenyl-2,5-dihydrofuran-3-yl)oxy)methyl)phenyl)acrylate* (**7d**). Yield: 42.1%; dark yellow solid; m.p.103–106 °C. ^1^H-NMR (600 MHz, chloroform-*d*) δ 7.92 (d, *J* = 7.2 Hz, 2H, ArH), 7.55 (s, 1H, CH_3_OCH), 7.46–7.41 (m, 1H, ArH), 7.43–7.34 (m, 4H, ArH), 7.29 (t, *J* = 7.4 Hz, 1H, ArH), 7.19 (d, *J* = 5.3 Hz, 1H, ArH), 5.11 (s, 2H, ArCH_2_), 4.70 (s, 2H, CH_2_), 3.74 (s, 3H, CH_3_OCH), 3.67 (s, 3H, CH_3_OCO). ^13^C-NMR (150 MHz, chloroform-*d*) δ 172.67, 172.54, 167.46, 160.63, 133.92, 131.78, 131.66, 129.52, 128.89, 128.62, 128.37, 127.83, 127.66, 127.51, 109.61, 103.15, 71.36, 64.98, 62.28, 51.94. MS (ESI) *m*/*z* 403.34 [M + Na]^+^, calcd. For C_22_H_20_O_6_ *m*/*z* = 380.13. ESI-HRMS: calcd. for C_22_H_20_O_6_ ([M + Na]^+^), 403.3804; found, 403.4203.

*Data for Methyl 2-(2-(((4-(2-chlorophenyl)-5-oxo-2,5-dihydrofuran-3-yl)oxy)methyl)phenyl)-3-methoxyacrylate* (**7e**). Yield: 23.7%; light yellow oil; ^1^H-NMR (600 MHz, chloroform-*d*) δ 7.47 (d, *J* = 7.3 Hz, 1H, CH_3_OCH), 7.44 (s, 1H, ArH), 7.40–7.27 (m, 6H, ArH), 7.17–7.10 (m, 1H, ArH), 4.88 (s, 2H, ArCH_2_), 4.71 (s, 2H, CH_2_), 3.71 (s, 3H, CH_3_OCH), 3.64 (s, 3H, CH_3_OCO). ^13^C-NMR (150 MHz, chloroform-*d*) δ 173.00, 172.78, 167.41, 160.31, 135.16, 133.68, 132.66, 131.77, 131.36, 130.08, 129.68, 129.41, 128.77, 128.36, 127.93, 126.85, 109.48, 102.02, 72.03, 66.97, 62.16, 51.84. MS (ESI) *m*/*z* 437.28 [M + Na]^+^, calcd. For C_22_H_19_ClO_6_ *m*/*z* = 414.09.

*Data for Methyl 2-(2-(((4-(4-chlorophenyl)-5-oxo-2,5-dihydrofuran-3-yl)oxy)methyl)phenyl)-3-methoxyacrylate* (**7f**). Yield: 30.2%; light yellow solid; m.p.149–152 °C. ^1^H-NMR (600 MHz, chloroform-*d*) δ 7.92 (dd, 2H, ArH), 7.57 (s, 1H, CH_3_OCH), 7.45–7.32 (m, 5H, ArH), 7.24–7.16 (m, 1H, ArH), 5.13 (s, 2H, ArCH_2_), 4.70 (s, 2H, CH_2_), 3.76 (s, 3H, CH_3_OCH), 3.67 (s, 3H, CH_3_OCO). ^13^C-NMR (150 MHz, chloroform-*d*) δ 172.86, 172.30, 167.42, 160.65, 133.70, 133.30, 131.89, 131.70, 131.06, 129.04, 128.87, 128.69, 128.56, 127.49, 109.63, 102.05, 71.48, 64.88, 62.32, 54.43, 51.97. MS (ESI) *m*/*z* 437.19 [M + Na]^+^, calcd. For C_22_H_19_ClO_6_ *m*/*z* = 414.09. ESI-HRMS: calcd. for C_22_H_19_ClO_6_ ([M + Na]^+^), 437.0768; found, 437.4438.

*Data for Methyl 2-(2-(((4-(2,6-dichlorophenyl)-5-oxo-2,5-dihydrofuran-3-yl)oxy)methyl)phenyl)-3-methoxyacrylate* (**7g**). Yield: 53.2%; light yellow solid; m.p.124–127 °C. ^1^H-NMR (600 MHz, chloroform-*d*) δ 7.44 (s, 1H, CH_3_OCH), 7.42–7.32 (m, 5H, ArH), 7.30 (t, *J* = 8.1 Hz, 1H, ArH), 7.15–7.12 (m, 1H, ArH), 4.85 (s, 2H, ArCH_2_), 4.76 (s, 2H, CH_2_), 3.73 (s, 3H, CH_3_OCH), 3.64 (s, 3H, CH_3_OCO). ^13^C-NMR (150 MHz, chloroform-*d*) δ 173.51, 171.45, 167.36, 160.33, 137.01, 133.31, 131.92, 131.33, 130.70, 129.14, 128.88, 128.37, 128.10, 128.02, 109.40, 99.98, 71.77, 67.29, 62.15, 51.83. MS (ESI) *m*/*z* 471.13 [M + Na]^+^, calcd. For C_22_H_18_Cl_2_O_6_ *m*/*z* = 448.05. ESI-HRMS: calcd. for C_22_H_18_Cl_2_O_6_ ([M + Na]^+^), 471.0378; found, 471.3771.

*Data for Methyl 3-methoxy-2-(2-(((4-(2-methoxyphenyl)-5-oxo-2,5-dihydrofuran-3-yl)oxy)methyl)phenyl)acrylate* (**7h**). Yield: 34.3%; yellow oil; ^1^H-NMR (600 MHz, chloroform-*d*) δ 7.40 (s, 1H, CH_3_OCH), 7.39–7.32 (m, 4H, ArH), 7.28 (dd, *J* = 7.5, 1.7 Hz, 1H, ArH), 7.14–7.11 (m, 1H, ArH), 7.00 (td, *J* = 7.5, 1.1 Hz, 1H, ArH), 6.94 (d, *J* = 8.3 Hz, 1H, ArH), 4.89 (s, 2H, ArCH_2_), 4.68 (s, 2H, CH_2_), 3.81 (s, 3H, ArOCH_3_), 3.64 (s, 3H, CH_3_OCH), 3.62 (s, 3H, CH_3_OCO). ^13^C-NMR (150 MHz, chloroform-*d*) δ 173.77, 172.63, 167.45, 160.25, 157.70, 134.27, 132.12, 131.47, 131.21, 130.03, 128.43, 128.24, 127.65, 120.63, 119.07, 111.08, 109.45, 100.39, 71.21, 66.96, 62.08, 55.61, 51.79. MS (ESI) *m*/*z* 433.32 [M + Na]^+^, calcd. For C_23_H_22_O_7_ *m*/*z* = 410.14.

*Data for Methyl 3-methoxy-2-(2-(((5-oxo-4-(p-tolyl)-2,5-dihydrofuran-3-yl)oxy)methyl)phenyl)acrylate* (**7i**). Yield: 38.6%; yellow solid; m.p.104–107 °C. ^1^H-NMR (600 MHz, chloroform-*d*) δ 7.81 (d, *J* = 8.2 Hz, 2H, ArH), 7.55 (s, 1H, CH_3_OCH), 7.43 (t, *J* = 4.7, 4.1 Hz, 1H, ArH), 7.42–7.34 (m, 2H, ArH), 7.19 (t, *J* = 8.9 Hz, 3H, ArH), 5.09 (s, 2H, ArCH_2_), 4.67 (s, 2H, CH_2_), 3.75 (s, 3H, CH_3_OCH), 3.67 (s, 3H, CH_3_OCO), 2.36 (s, 3H, ArCH_3_). ^13^C-NMR (150 MHz, chloroform-*d*) δ 172.81, 171.93, 167.47, 160.61, 137.49, 134.04, 131.74, 131.59, 129.08, 128.84, 128.62, 127.74, 127.45, 126.60, 109.64, 103.23, 71.22, 64.99, 62.28, 51.94, 21.46. MS (ESI) *m*/*z* 417.29 [M + Na]^+^, calcd. For C_23_H_22_O_6_ *m*/*z* = 394.14. ESI-HRMS: calcd. for C_23_H_22_O_6_ ([M + Na]^+^), 417.4069; found, 417.5518.

*Data for Methyl 2-(2-(((4-(2-fluorophenyl)-5-oxo-2,5-dihydrofuran-3-yl)oxy)methyl)phenyl)-3-methoxyacrylate* (**7j**). Yield:41.3%; light yellow solid; m.p.102–105 °C. ^1^H-NMR (600 MHz, chloroform-*d*) δ 7.49 (s, 1H, CH_3_OCH), 7.47–7.37 (m, 2H, ArH), 7.40–7.31 (m, 3H, ArH), 7.24–7.17 (m, 1H, ArH), 7.19–7.09 (m, 2H, ArH), 4.99 (s, 2H, ArCH_2_), 4.69 (s, 2H, CH_2_), 3.73 (s, 3H, CH_3_OCH), 3.65 (s, 3H, CH_3_OCO). ^13^C-NMR (150 MHz, chloroform-*d*) δ 173.66, 172.70, 167.43, 160.43 (d, ^1^*J_C-F_* = 247.5 Hz), 160.35, 133.81, 131.98 (d, ^4^*J_C-F_* = 3 Hz), 131.63, 131.47, 130.32 (d, ^3^*J_C-F_* = 7.5 Hz), 128.77, 128.47, 127.75, 124.22 (d, ^4^*J_C-F_* = 3 Hz), 117.55 (d, ^2^*J_C-F_* = 15 Hz), 115.80 (d, ^2^*J_C-F_* = 21 Hz), 109.54, 98.64, 71.62, 66.56, 62.18, 51.86. MS (ESI) *m*/*z* 421.26 [M + Na]^+^, calcd. For C_22_H_19_FO_6_ *m*/*z* = 398.12. ESI-HRMS: calcd. for C_22_H_19_FO_6_ [M + Na]^+^, 421.3708; found, 421.5121.

*Data for Methyl 3-methoxy-2-(2-(((5-oxo-4-(2,4,5-trifluorophenyl)-2,5-dihydrofuran-3-yl)oxy)methyl)phenyl)acrylate* (**7k**). Yield: 33.2%; yellow solid; m.p. 92–95 °C. ^1^H-NMR (600 MHz, chloroform-*d*) δ 7.55 (s, 1H, CH_3_OCH), 7.41–7.35 (m, 3H, ArH), 7.32–7.27 (m, 1H, ArH), 7.19–7.16 (m, 1H, ArH), 7.03–6.97 (m, 1H, ArH), 5.04 (s, 2H, ArCH_2_), 4.69 (s, 2H, CH_2_), 3.80 (s, 3H, CH_3_OCH), 3.68 (s, 3H, CH_3_OCO). ^13^C-NMR (150 MHz, chloroform-*d*) δ 174.38, 171.92, 167.36, 160.45, 133.53, 131.66, 131.56, 131.45, 128.92, 128.62, 127.44, 119.31 (d, *J_C-F_* = 6 Hz), 119.18 (d, *J_C-F_* = 4.5 Hz), 109.59, 105.96 (d, *J_C-F_* = 27 Hz), 105.82 (d, *J_C-F_* = 27 Hz), 97.45, 71.61, 66.25, 62.29, 51.91. MS (ESI) *m*/*z* 457.17 [M + Na]^+^, calcd. For C_22_H_17_F_3_O_6_ *m*/*z* = 434.10.

### 4.3. Biological Activity Assay

All fungal phytopathogenic strains were obtained from the Hubei Biopesticide Engineering Research Center at the Hubei Academy of Agricultural Sciences and College of Plant Science and Technology of Huazhong Agricultural University. Commercial fungicides Azoxystrobin and Carbendazim as a positive fungicide control for bioassay were bought from Aladdin Reagent Database Co. (Shanghai, China).

All compounds were tested for common phytopathogenic fungi from six major crops, mainly including tea, rape, peach, wheat, rice and pepper, to control the *Phomopsis adianticola, Rap Sclerotinia stem rot, Pestallozzia theae, Gibberella zeae*, *Sclerotinia sclerotiorum*, *Altermaria alternate*, *Pestalotiopsis theae*, *Monilinia fructicola*, *Phytophthora capsica*, *Magnapothe grisea*.

The growth inhibition rates were calculated by means of the following equation: I = [(D_0_ − D_t_)/D_0_] × 100%, where I is the growth inhibition rate (%), D_0_ is the blank control colony growth diameter (mm) and D_t_ is the pharmacy treatment colony growth diameter (mm) [21,22,23].

#### 4.3.1. Primary Screening Test for Inhibiting Mycelial Growth of Pathogenic Fungi

Each of the test compounds (25 mg) was first dissolved in 2.5 mL acetone to generate a 10^4^ mg·L^−1^ stock solution, then the stock solution was added to 250 mL of Potato Dextrose Agar (Medium) that is PDA to prepare a drug-loaded PDA medium plate having a concentration of 100 mg·L^−1^, and only 2.5 mL acetone was added to the PDA as a blank control, Azoxystrobin and Carbendazim as positive fungicides control. The above 12 pathogens were activated (prepared by a punch with Φ = 0.50 cm), and they were cut from the edge of the colony, inoculated into the above PDA plate, and each strain and blank control were repeated twice, sealing with a sealing film to prevent contamination of other bacteria, the poisonous PDA culture dishes were placed in a constant temperature incubator at the temperature condition of 30 ± 1 °C. After the blank control group culture was grown to about two-thirds of the culture dish, the colony diameter was measured by the cross method in millimeters (mm) with a cross and a vertical method, and the average value was taken, and the inhibition rate of each compound against the fungus was calculated.

#### 4.3.2. Further Screening Test for Inhibiting Mycelial Growth of Pathogenic Fungi

According to the results of the primary screening test, compounds **7e** and **7k** with good inhibition rates and 4 strains were selected for a further screening test for inhibiting mycelial growth of pathogenic fungi in the lower concentration range sequentially. The method used is still the same as the primary screening. The difference is that the selected compounds have five different concentration gradients for the growth inhibition of each strain, that is, 100, 50, 25, 12.5, and 6.25 mg·L^−1^.

## Figures and Tables

**Table 1:** Preliminary inhibitory effect of target compounds and intermediates on twelve common plant pathogenic fungi (100 mg·L^−1^).

Antifungal Activities In Vitro (100 µg·mL^−1^, Inhibitory Rate %)												
Compd. No.	*Rhizoctonia solani*	*Phomopsis adianticola*	*Rap Sclerotinia stem rot*	*Pestallozzia theae*	*Gibberella zeae*	*Sclerotinia sclerotiorum*	*Altermaria alternata*	*Pestalotiopsis theae*	*Monilinia fructicola*	*Phytophthora capsici*	*Colletotrichum gloeosporioides*	*Magnapothe grisea*
**6c**	- ^a^	7.9	12.24	12.27	/	4.12	7.08	37.79	16.70	/	-	7.87
**6i**	-	13.75	41.49	34.33	17.65	51.03	21.77	47.16	64.09	10.25	-	28.32
**7a**	/ ^b^	64.43	72.84	33.17	/	14.71	9.56	0.33	35.91	/	/	25.52
**7b**	32.31	58.25	74.18	48.92	16.34	43.82	20.18	33.61	37.88	/	5.61	38.46
**7c**	-	43.64	73.28	37.81	29.20	20.29	20.00	19.73	36.09	2.65	-	29.37
**7d**	1.15	43.64	73.28	45.94	23.20	12.94	20.00	30.77	41.47	24.91	2.46	29.72
**7e**	46.73	66.15	72.99	49.25	45.10	24.26	21.59	23.41	52.42	13.60	7.72	27.62
**7f**	23.85	38.14	35.07	33.00	29.90	13.53	19.82	/	24.06	17.31	3.86	29.55
**7g**	/	86.94	20.45	22.06	28.76	19.12	17.52	/	9.16	/	/	38.81
**7h**	/	29.55	13.43	37.31	14.87	8.53	21.42	38.13	30.52	/	4.21	28.67
**7i**	-	36.08	31.94	36.65	32.68	10.88	27.08	8.36	36.80	/	-	26.05
**7j**	/	55.50	27.31	39.64	5.07	6.32	17.88	34.28	33.57	19.08	5.96	24.48
**7k**	1.92	51.03	73.58	45.11	44.28	17.94	23.19	32.27	33.93	10.60	10.18	38.11
Intermediate I	33.08	73.88	60.60	63.18	66.99	97.65	39.82	60.03	77.74	0.00	21.93	29.02
Azoxystrobin	88.65	95.19	93.43	100.00	68.63	100.00	59.65	100.00	93.00	24.03	58.95	100.00
Carbendazim	100.00	100.00	99.40	100.00	100.00	100.00	17.70	100.00	100.00	6.36	100.00	100.00

^a^ denotes that the compound was not tested. ^b^ denotes that the compound has no inhibitory activity.

**Table 2:** In vitro antifungal activities of compounds **7k** and **7e** on four common plant pathogenic fungi.

Compd. No.	Concentration	*Phomopsis adianticola*	*Pestallozzia theae*	*Gibberella zeae*	*Sclerotinia sclerotiorum*
**7k**	100	36.91	39.14	46.62	33.14
	50	36.76	38.97	10.44	27.44
	25	29.71	37.93	12.66	23.32
	12.5	30.15	31.03	7.47	12.07
	6.25	4.12	20.00	3.91	8.90
**7e**	100	35.44	42.59	/ ^a^	43.28
	50	35.00	40.17	22.60	44.07
	25	28.38	22.59	5.99	50.10
	12.5	13.53	24.31	/	11.28
	6.25	10.59	14.66	1.54	19.04
Carbendazim	100	100.00	100.00	100.00	100.00
	50	50.00	100.00	87.69	100.00
	25	100.00	100.00	89.77	100.00
	12.5	100.00	100.00	89.92	100.00
	6.25	100.00	100.00	82.21	100.00
Intermediate I	100	69.26	32.76	1.84	26.96
	50	41.76	23.62	/	14.29
	25	30.29	17.07	/	/
	12.5	12.94	/	/	/
	6.25	21.18	6.38	/	/

^a^ denotes that the compound has no inhibitory activity.

## Supplementary Materials

The following are available online. ^1^H-NMR, ^13^C-NMR, MS of the target compounds.
