# Supplementary material for: Synthesis and Bio-Evaluation of Natural Butenolides-Acrylate Conjugates
Source: Molecules. 2019 Apr 3;24(7):1304. doi: 10.3390/molecules24071304 (PMC6480375; doi:10.3390/molecules24071304)
Supplement: Supplementary file 1 [file molecules-24-01304-s001.pdf]

Synthesis and Bio-Evaluation of Natural Butenolides-Acrylate Conjugates

Longzhu Bao [1], Shuangshuang Wang [1], Di Song [1], Jingjing Wang [1], Xiufang Cao [1,*] and Shaoyong Ke [2,*]

[1] College of Science, Huazhong Agricultural University, Wuhan 430070, China; baolz@webmail.hzau.edu.cn (L.B.); 1140291490@webmail.hzau.edu.cn (S.W.); songdi@webmail.hzau.edu.cn (D.S.); 13523041961@163.com (J.W.)

[2] National Biopesticide Engineering Research Center, Hubei Biopesticide Engineering Research Center, Hubei Academy of Agricultural Science, Wuhan 430064, China

* Correspondence: caoxiufang@mail.hzau.edu.cn (X.C.); shaoyong.ke@nberc.com (S.K.); Tel.: +027-5910-1919 (S.K.)

Table of Contents

Spectral data for Methyl 3-methoxy-2-(2-(((5-oxo-4-(o-tolyl)-2,5-dihydrofuran-3-yl)oxy)methyl)phenyl)acrylate (7a). I

Spectral data for Methyl 2-(2-(((4-(2,4-dichlorophenyl)-5-oxo-2,5-dihydrofuran-3-yl)oxy)methyl)phenyl)-3-methoxyacrylate (7b). II

Spectral data for Methyl 2-(2-(((4-(4-fluorophenyl)-5-oxo-2,5-dihydrofuran-3-yl)oxy)methyl)phenyl)-3-methoxyacrylate (7c). IV

Spectral data for Methyl 3-methoxy-2-(2-(((5-oxo-4-phenyl-2,5-dihydrofuran-3-yl)oxy)methyl)phenyl)acrylate (7d). V

Spectral data for Methyl 2-(2-(((4-(2-chlorophenyl)-5-oxo-2,5-dihydrofuran-3-yl)oxy)methyl)phenyl)-3-methoxyacrylate (7e). VII

Spectral data for Methyl 2-(2-(((4-(4-chlorophenyl)-5-oxo-2,5-dihydrofuran-3-yl)oxy)methyl)phenyl)-3-methoxyacrylate (7f). VIII

Spectral data for Methyl 2-(2-(((4-(2,6-dichlorophenyl)-5-oxo-2,5-dihydrofuran-3-yl)oxy)methyl)phenyl)-3-methoxyacrylate (7g). X

Spectral data for Methyl 3-methoxy-2-(2-(((4-(2-methoxyphenyl)-5-oxo-2,5-dihydrofuran-3-yl)oxy)methyl)phenyl)acrylate (7h). XI

Spectral data for Methyl 3-methoxy-2-(2-(((5-oxo-4-(p-tolyl)-2,5-dihydrofuran-3-yl)oxy)methyl)phenyl)acrylate (7i). XIII

Spectral data for Methyl 2-(2-(((4-(2-fluorophenyl)-5-oxo-2,5-dihydrofuran-3-yl)oxy)methyl)phenyl)-3-methoxyacrylate (7j). XIV

Spectral data for Methyl 3-methoxy-2-(2-(((5-oxo-4-(2,4,5-trifluorophenyl)-2,5-dihydrofuran-3-yl)oxy)methyl)phenyl)acrylate (7k). XVI

Spectral data for Methyl 3-methoxy-2-(2-(((5-oxo-4-(o-tolyl)-2,5-dihydrofuran-3-yl)oxy)methyl)phenyl)acrylate (7a).

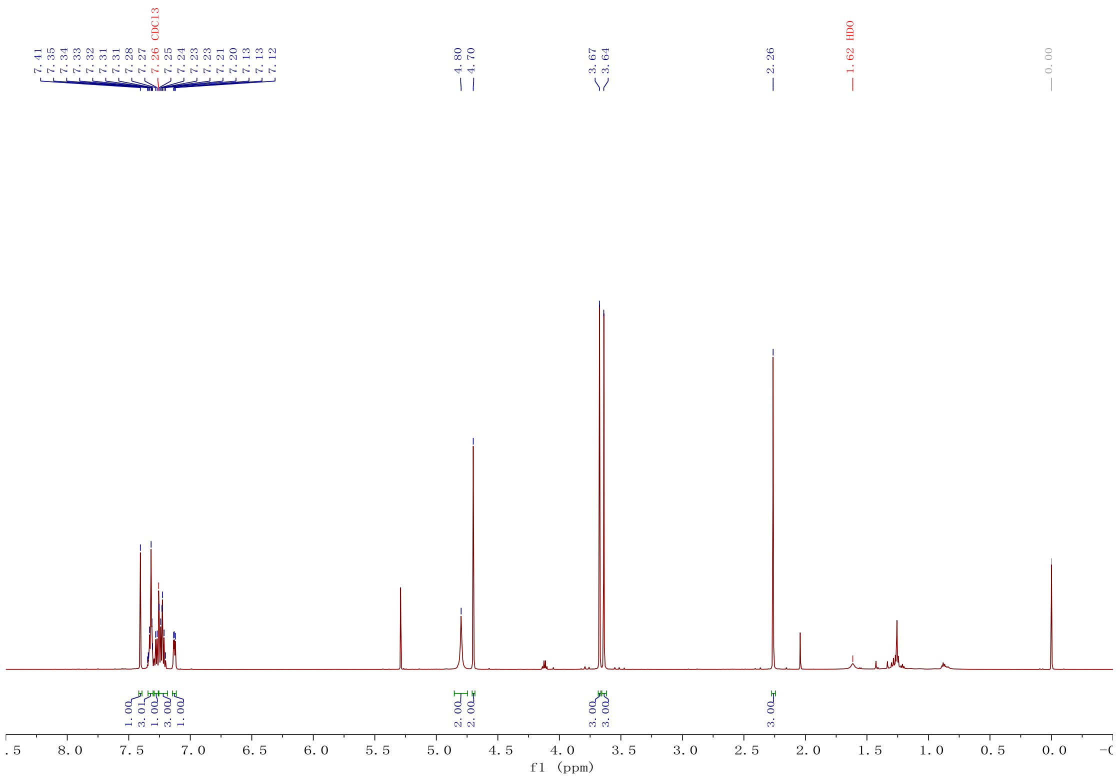

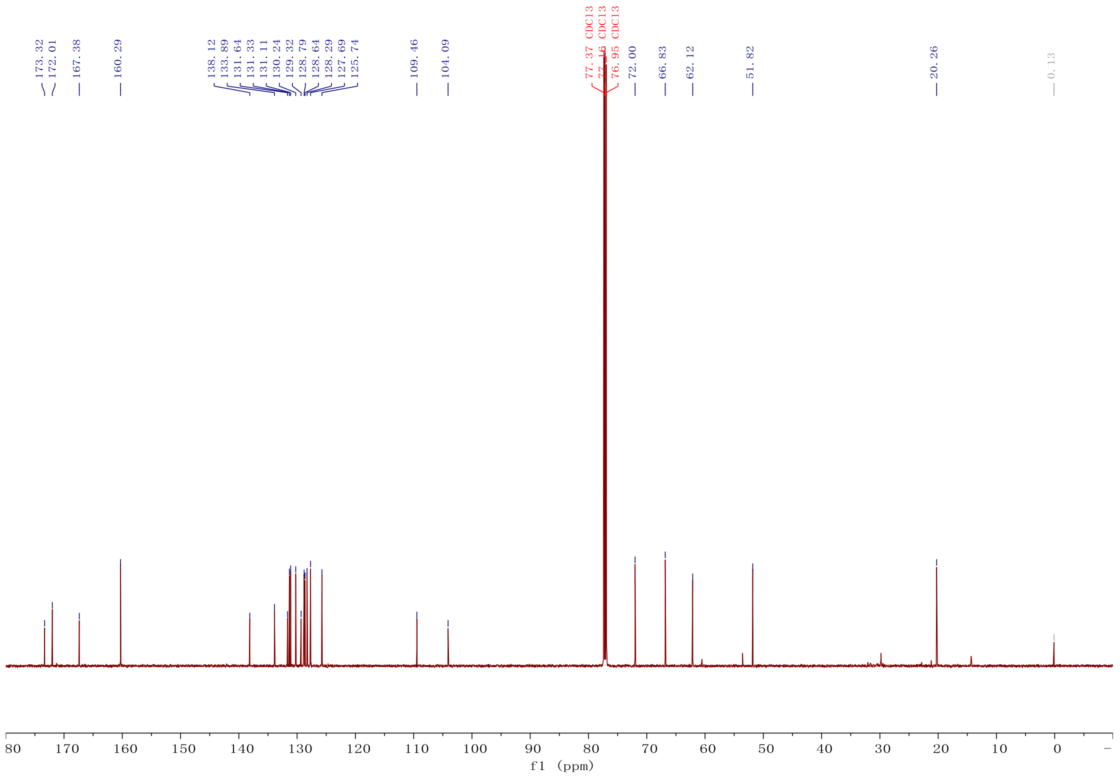

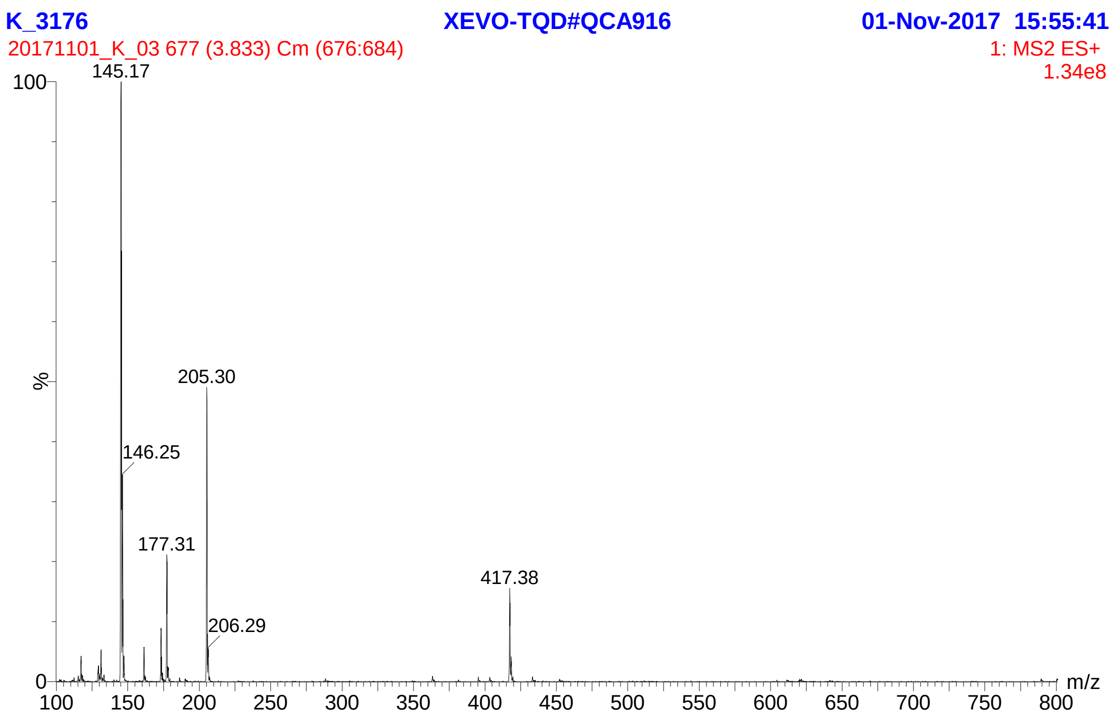

Spectral data for Methyl 2-(2-(((4-(2,4-dichlorophenyl)-5-oxo-2,5-dihydrofuran-3-yl)oxy)methyl)phenyl)-3-methoxyacrylate (7b).

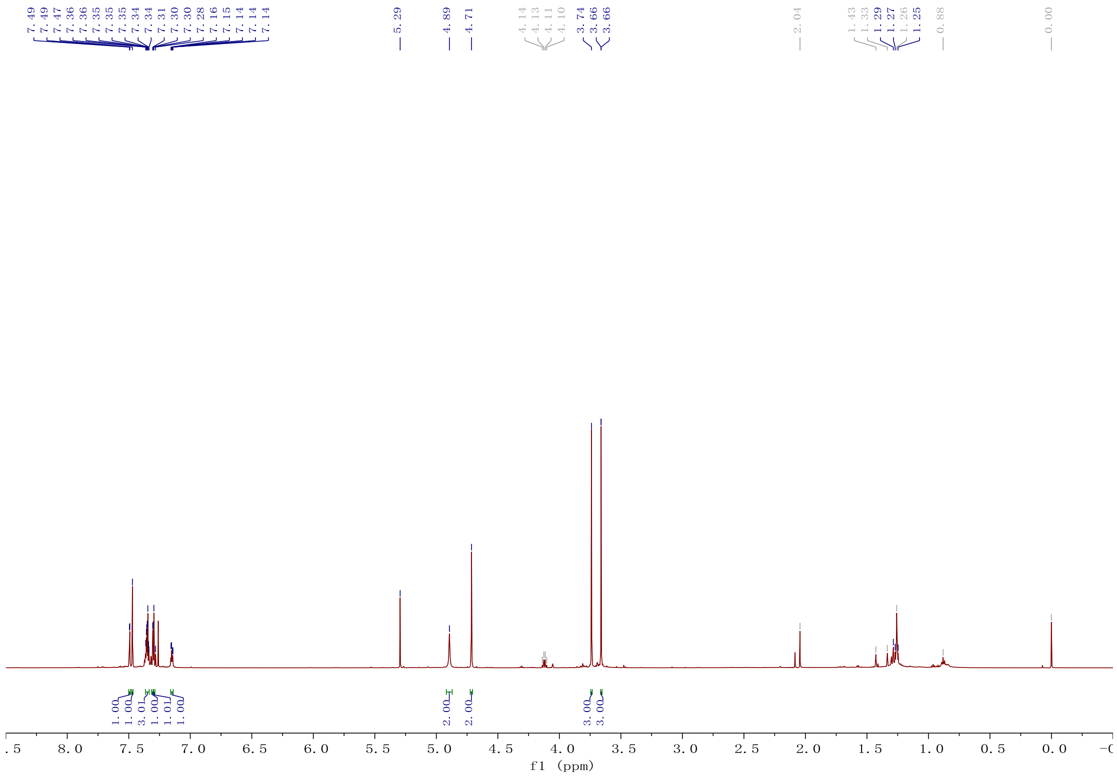

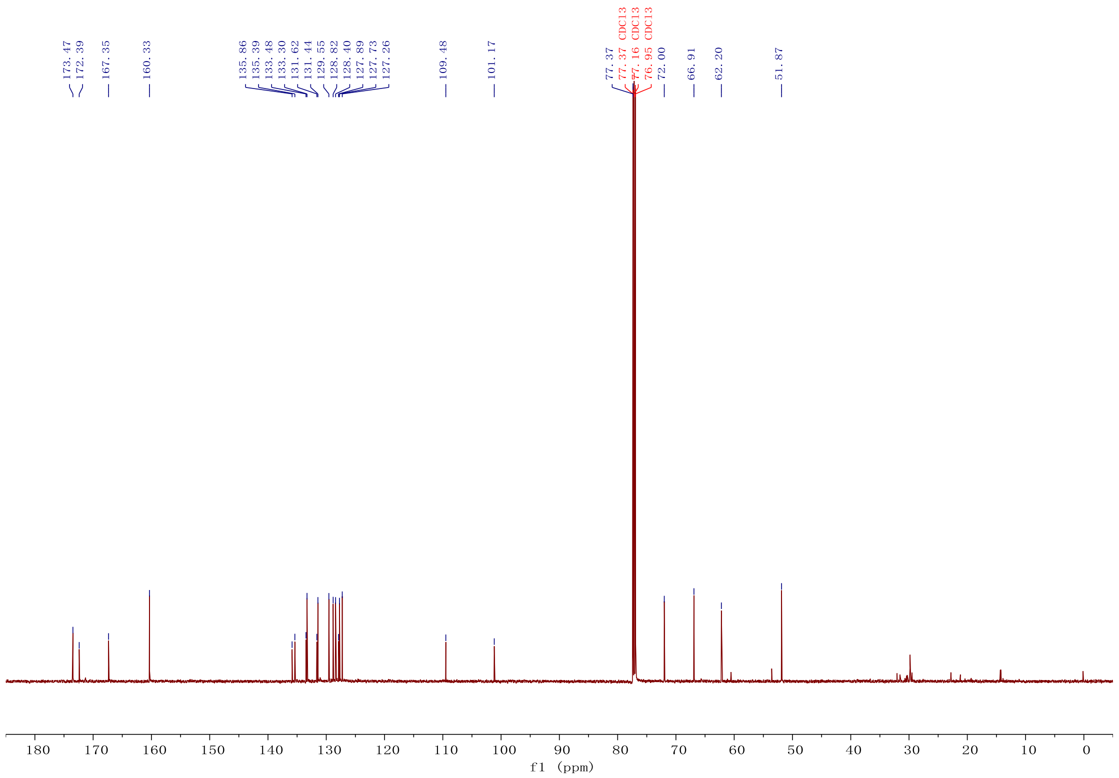
173.47
172.39
167.35
160.33
135.86
135.39
133.48
133.30
131.62
131.44
129.55
128.82
128.40
127.89
127.73
127.26
109.48
101.17
77.37
77.37 CDCl3
77.16 CDCl3
76.95 CDCl3
72.00
66.91
62.20
51.87
f1 (ppm)

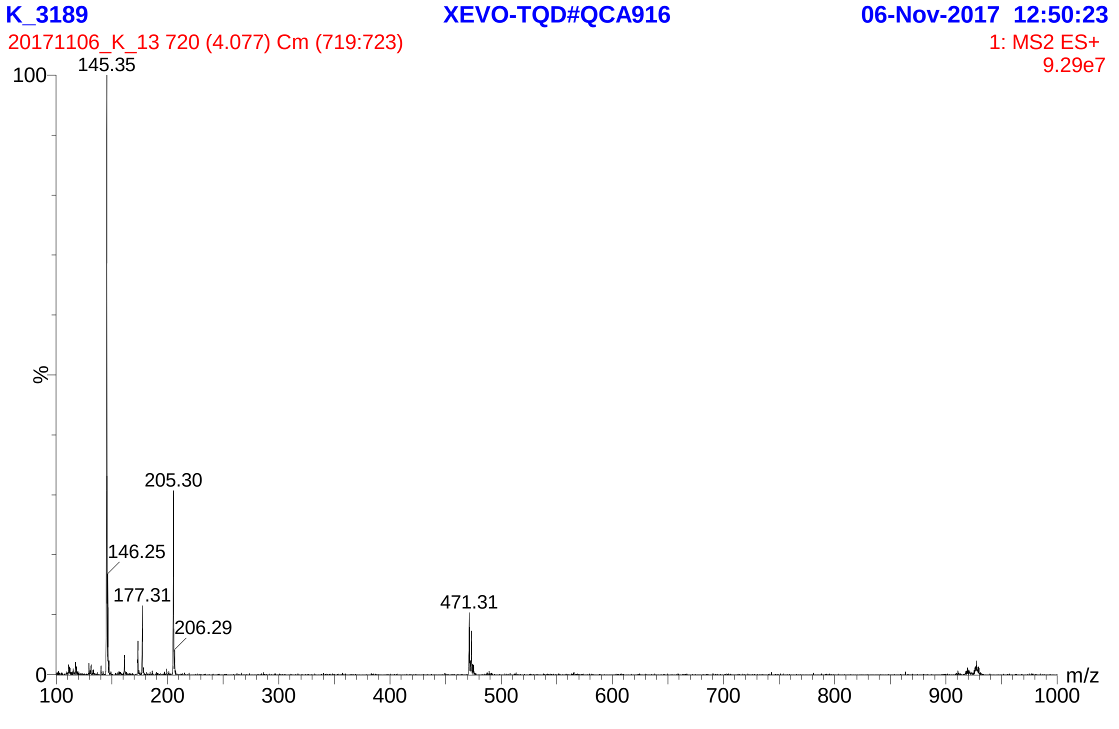
K_3189
XEVO-TQD#QCA916
06-Nov-2017 12:50:23
20171106_K_13 720 (4.077) Cm (719:723)
1: MS2 ES+
9.29e7
145.35
205.30
146.25
177.31
206.29
471.31
%
m/z

Spectral data for Methyl 2-(2-(((4-(4-fluorophenyl)-5-oxo-2,5-dihydrofuran-3-yl)oxy)methyl)phenyl)-3-methoxyacrylate (7c).

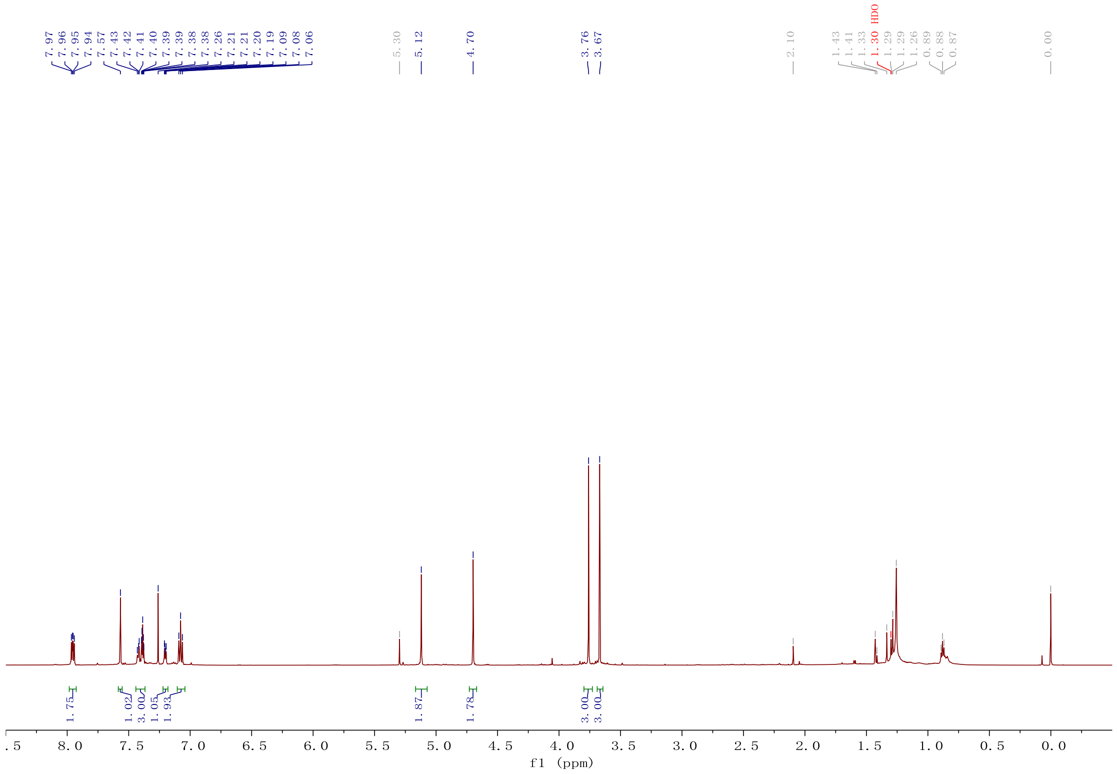

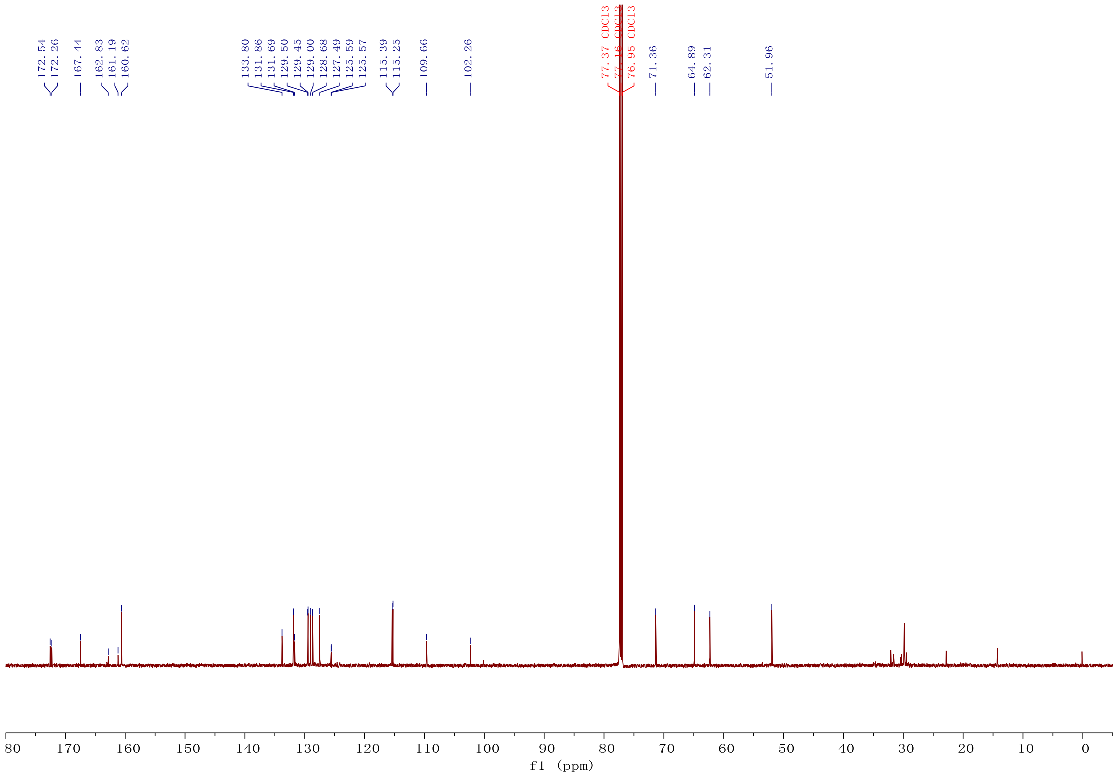

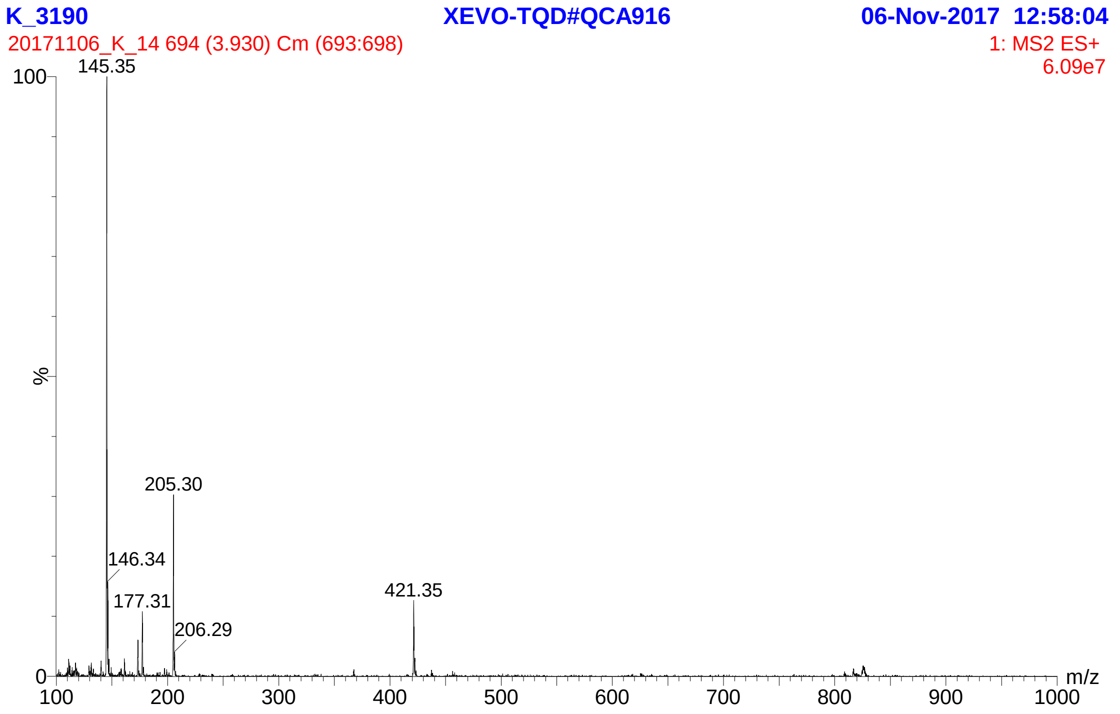

Spectral data for Methyl 3-methoxy-2-(2-(((5-oxo-4-phenyl-2,5-dihydrofuran-3-yl)oxy)methyl)phenyl)acrylate (7d).

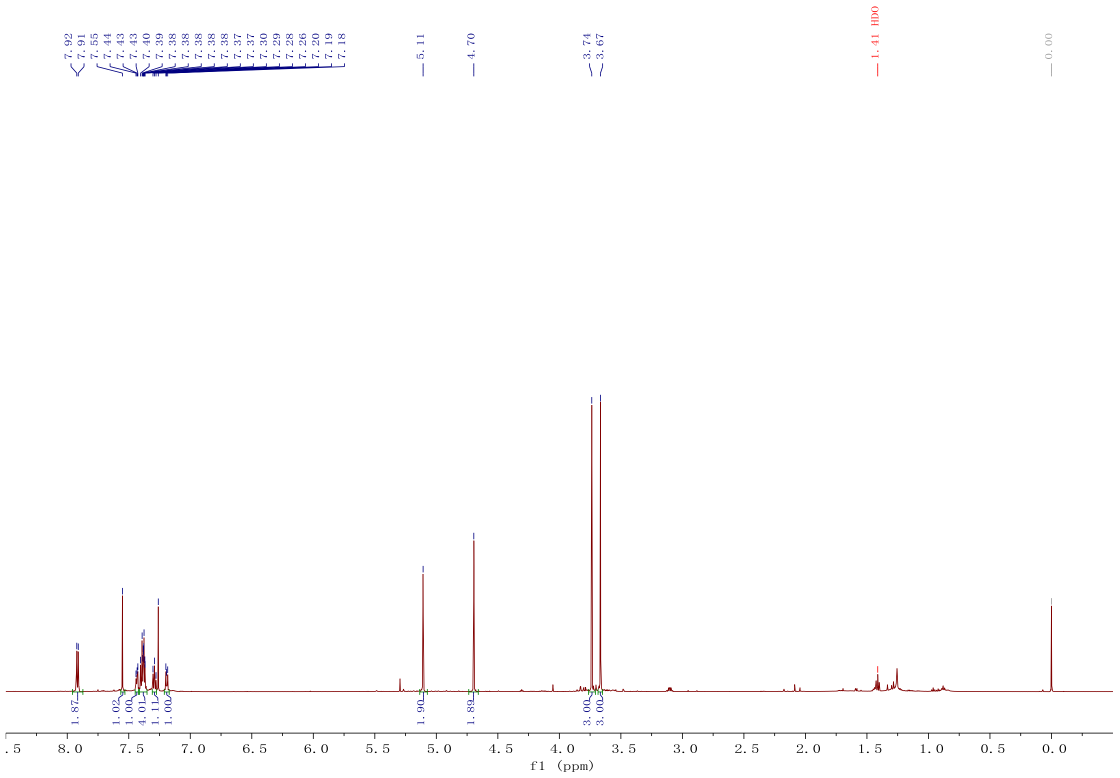

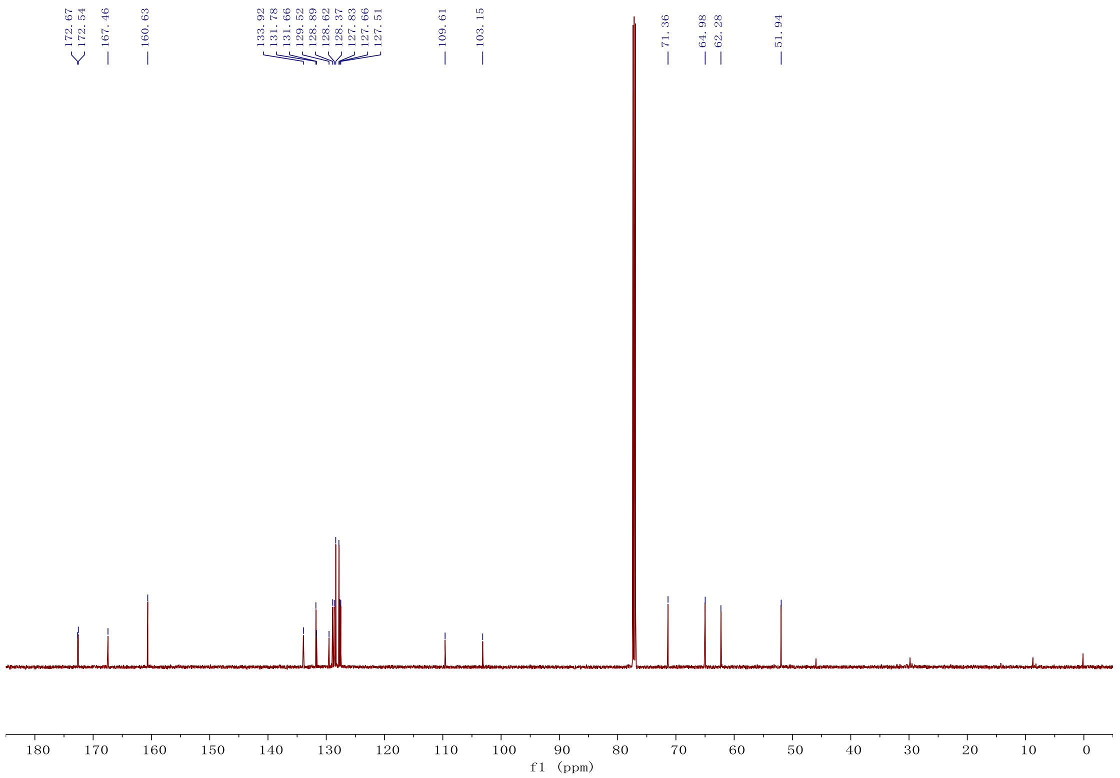
172.67
172.54
167.46
160.63
133.92
131.78
131.66
129.52
128.89
128.62
128.37
127.83
127.66
127.51
109.61
103.15
71.36
64.98
62.28
51.94
180 170 160 150 140 130 120 110 100 90 80 70 60 50 40 30 20 10 0
f1 (ppm)

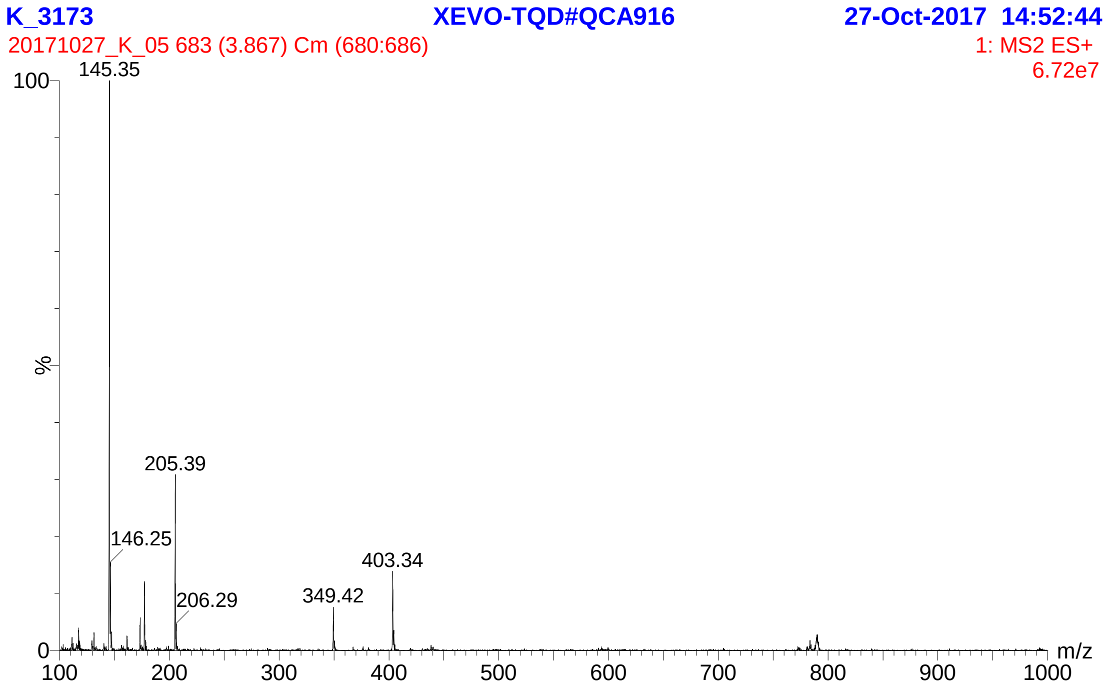
K_3173
XEVO-TQD#QCA916
27-Oct-2017 14:52:44
20171027_K_05 683 (3.867) Cm (680:686)
1: MS2 ES+
6.72e7
145.35
205.39
146.25
206.29
349.42
403.34
100
%
0
m/z
100 200 300 400 500 600 700 800 900 1000

Spectral data for Methyl 2-(2-(((4-(2-chlorophenyl)-5-oxo-2,5-dihydrofuran-3-yl)oxy)methyl)phenyl)-3-methoxyacrylate (7e).

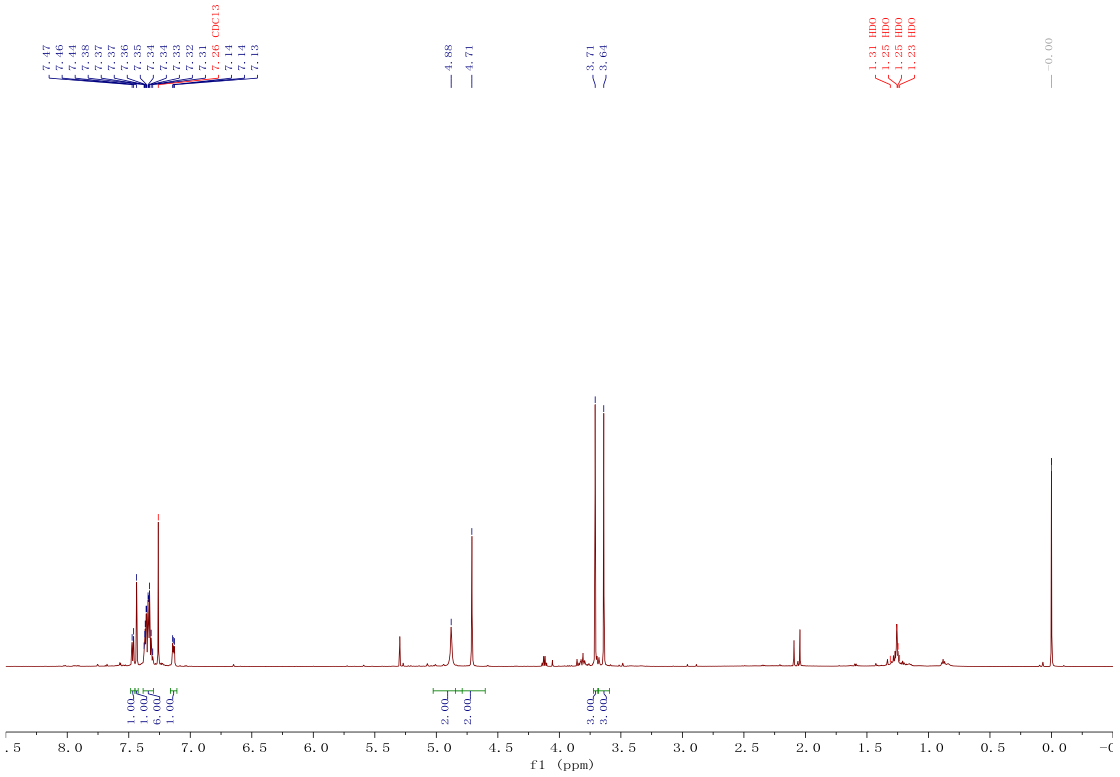

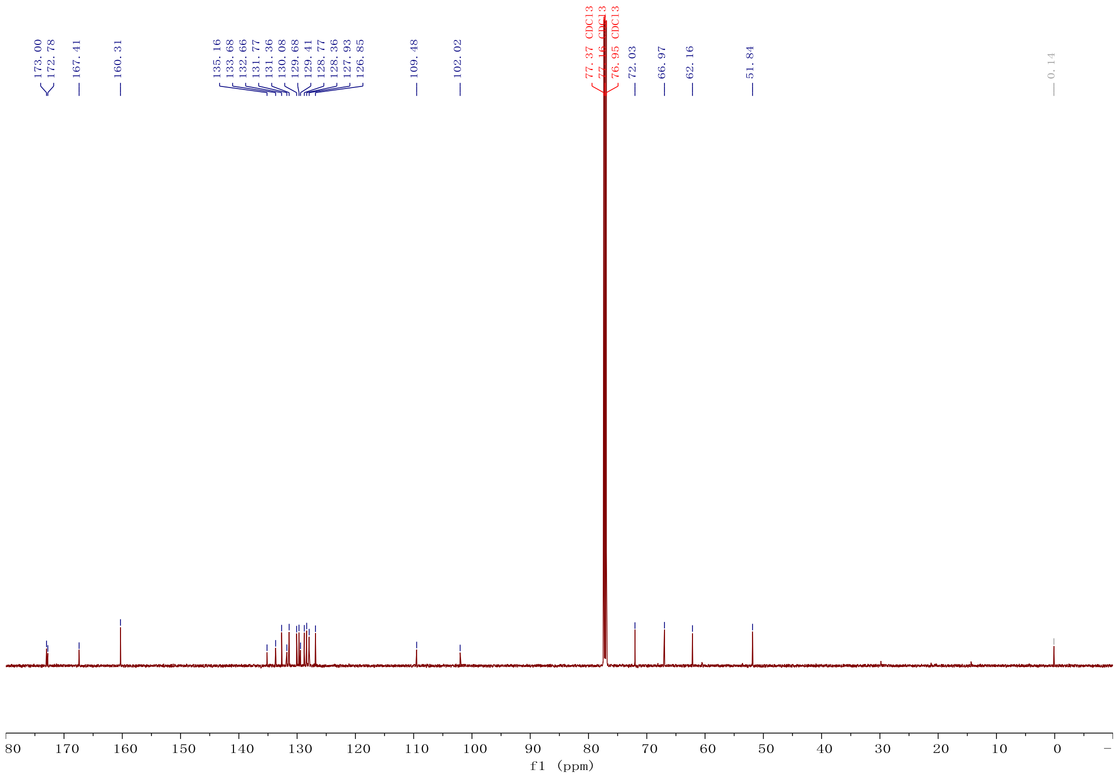

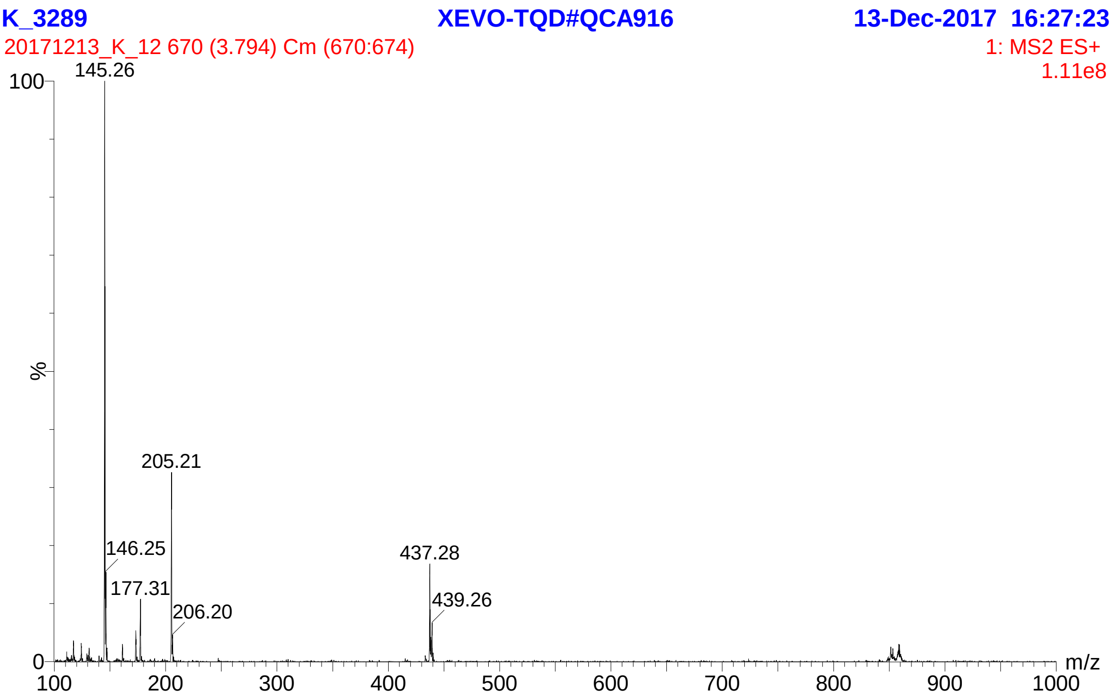

Spectral data for Methyl 2-(2-(((4-(4-chlorophenyl)-5-oxo-2,5-dihydrofuran-3-yl)oxy)methyl)phenyl)-3-methoxyacrylate (7f).

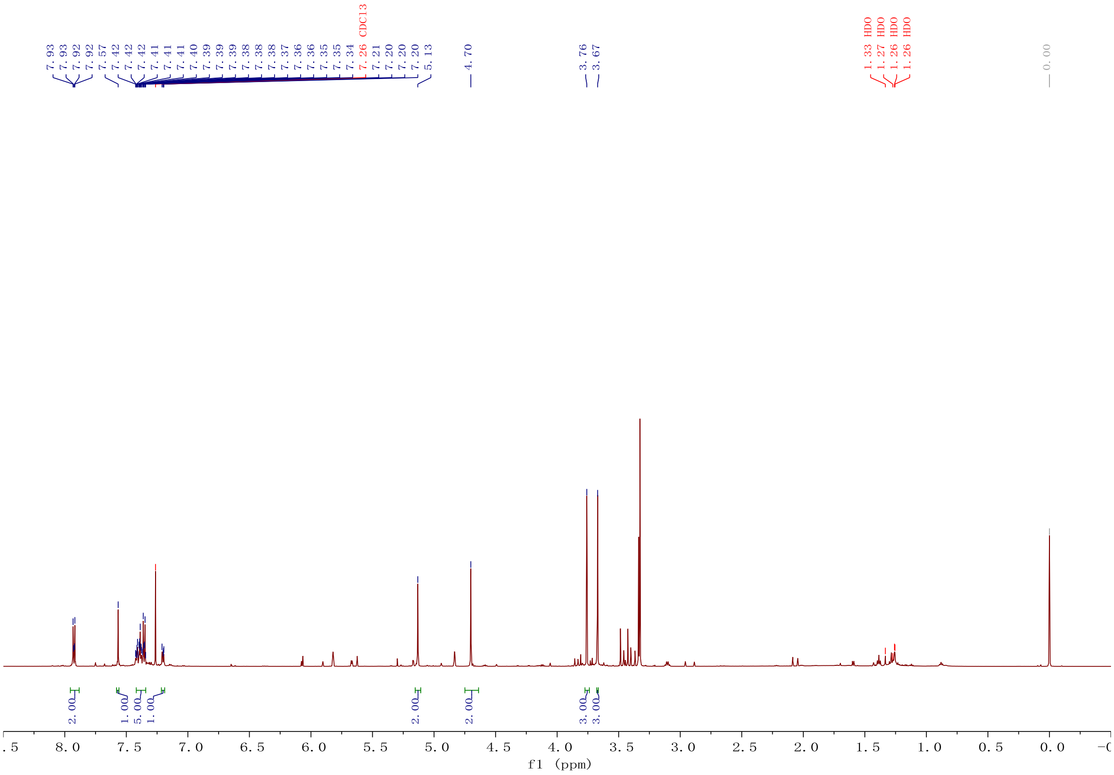

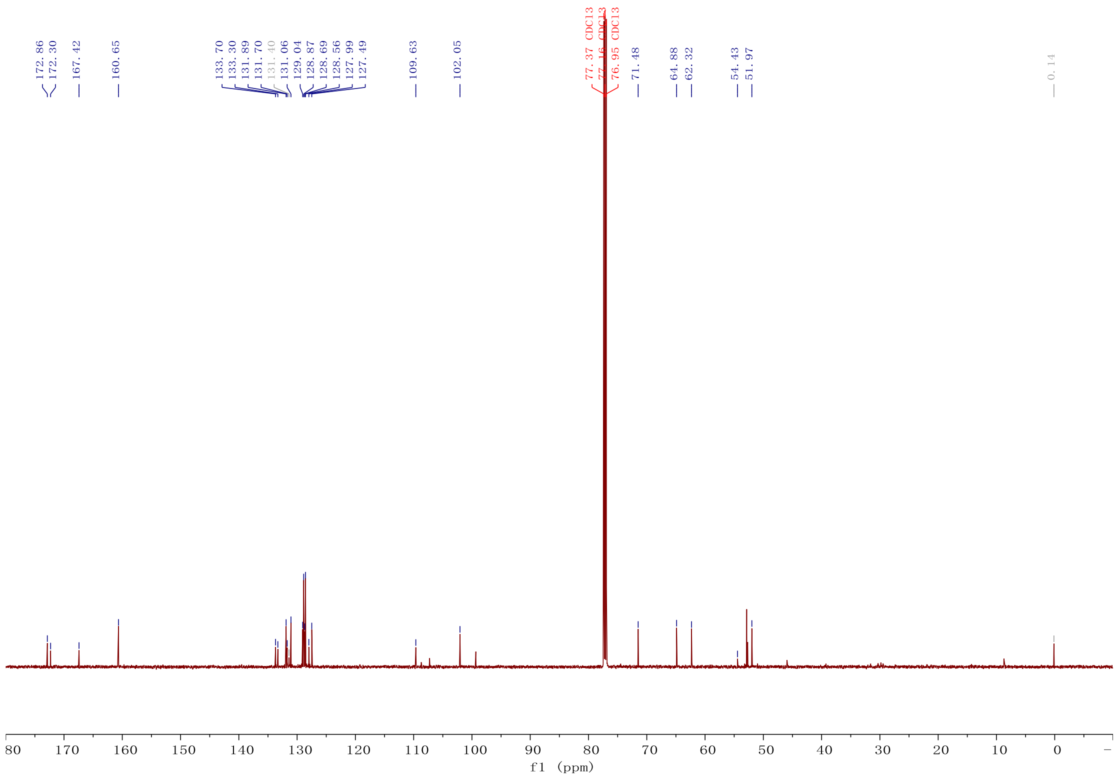
172.86
172.30
167.42
160.65
133.70
133.30
131.89
131.70
131.40
131.06
129.04
128.87
128.69
128.56
127.99
127.49
109.63
102.05
77.37 CDCl3
76.95 CDCl3
71.48
64.88
62.32
54.43
51.97
0.14
f1 (ppm)

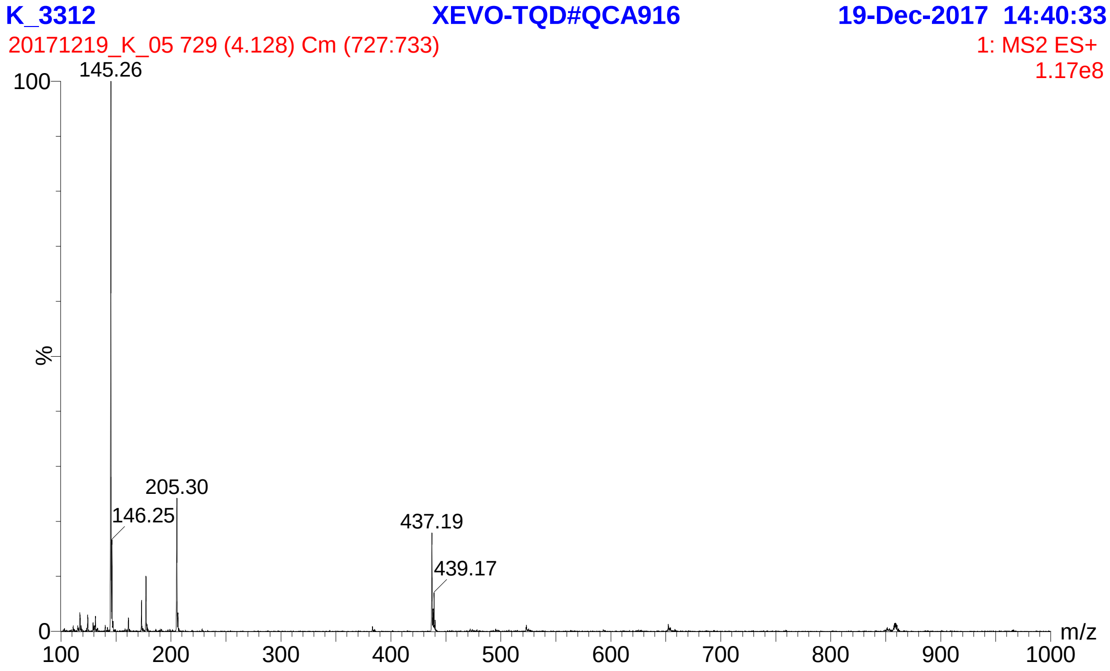
K_3312
XEVO-TQD#QCA916
19-Dec-2017 14:40:33
20171219_K_05 729 (4.128) Cm (727:733)
1: MS2 ES+
1.17e8
145.26
146.25
205.30
437.19
439.17
%
m/z

Spectral data for Methyl 2-(2-(((4-(2,6-dichlorophenyl)-5-oxo-2,5-dihydrofuran-3-yl)oxy)methyl)phenyl)-3-methoxyacrylate (7g).

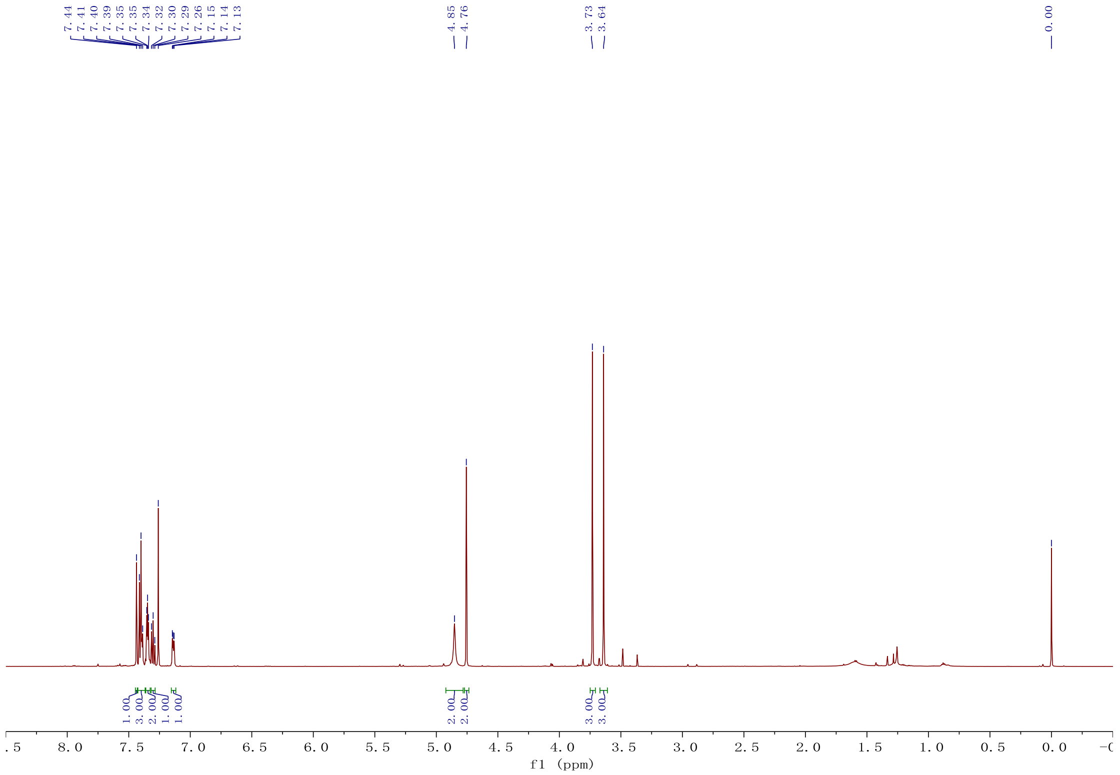

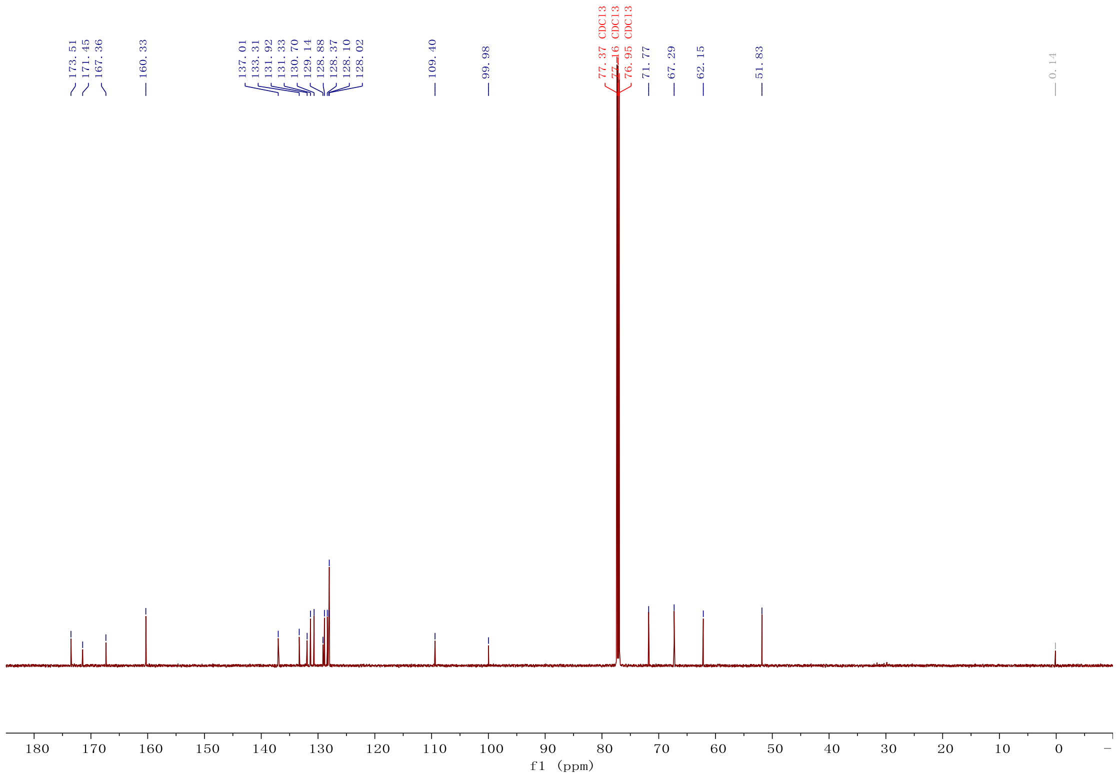

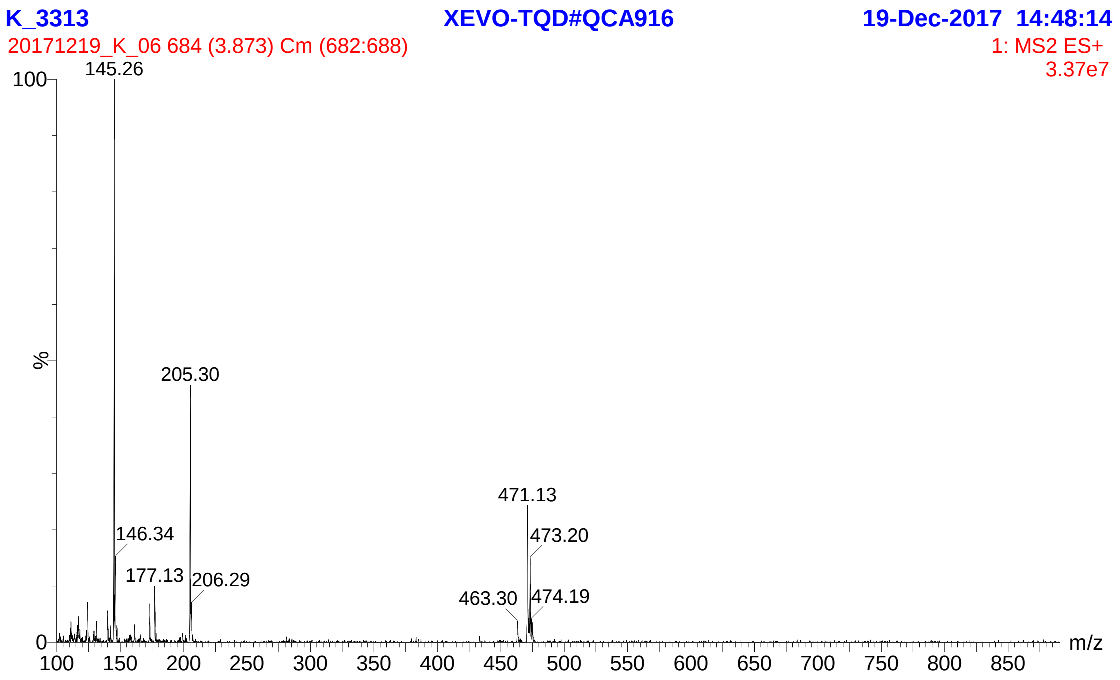

Spectral data for Methyl 3-methoxy-2-(2-(((4-(2-methoxyphenyl)-5-oxo-2,5-dihydrofuran-3-yl)oxy)methyl)phenyl)acrylate (7h).

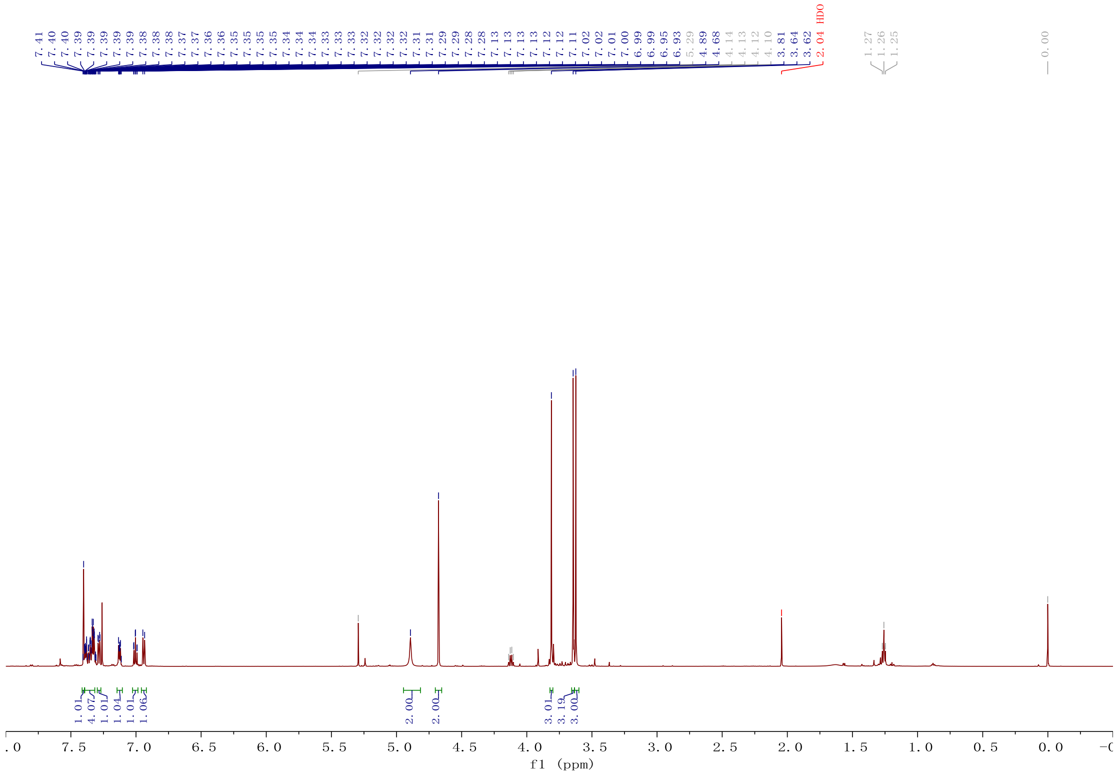

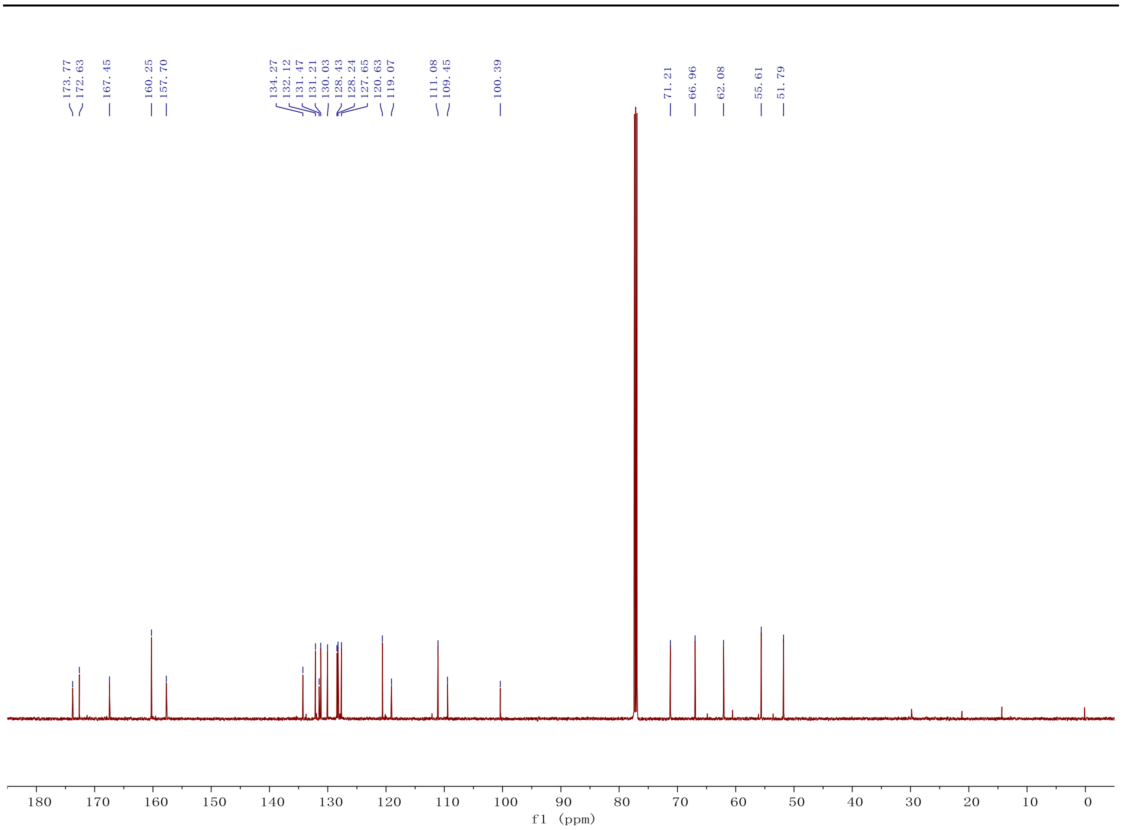
173.77
172.63
167.45
160.25
157.70
134.27
132.12
131.47
131.21
130.03
128.43
128.24
127.65
120.63
119.07
111.08
109.45
100.39
71.21
66.96
62.08
55.61
51.79
180 170 160 150 140 130 120 110 100 90 80 70 60 50 40 30 20 10 0
f1 (ppm)

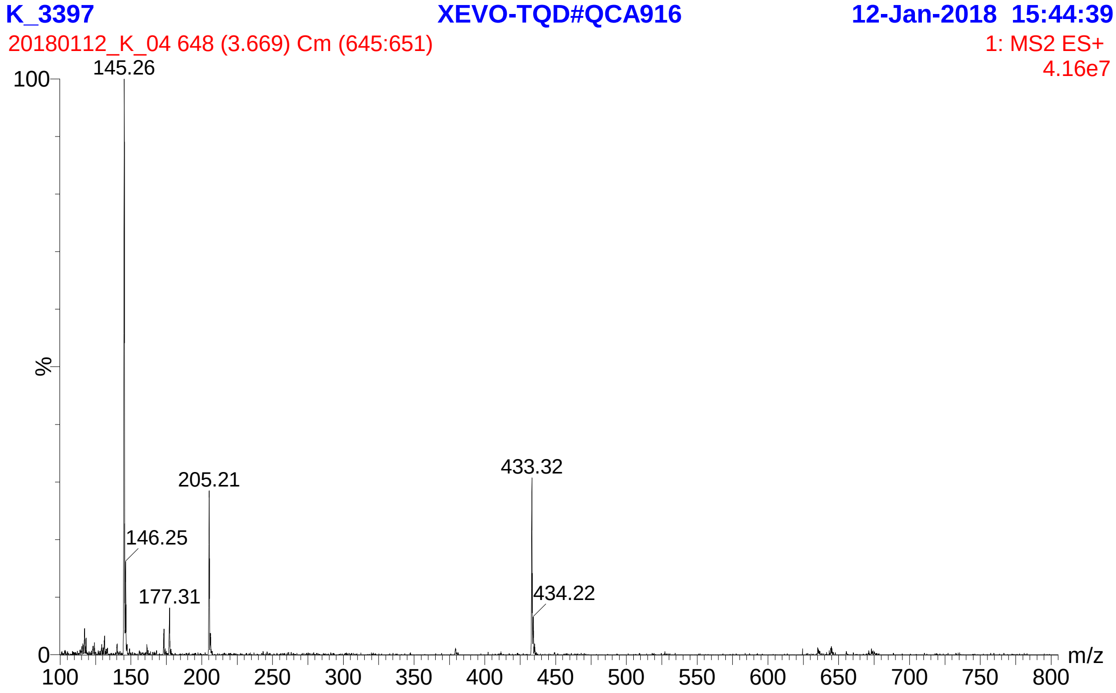
K_3397
XEVO-TQD#QCA916
12-Jan-2018 15:44:39
20180112_K_04 648 (3.669) Cm (645:651)
1: MS2 ES+
4.16e7
100
%
0
145.26
146.25
177.31
205.21
433.32
434.22
100 150 200 250 300 350 400 450 500 550 600 650 700 750 800
m/z

Spectral data for Methyl 3-methoxy-2-(2-(((5-oxo-4-(p-tolyl)-2,5-dihydrofuran-3-yl)oxy)methyl)phenyl)acrylate (7i).

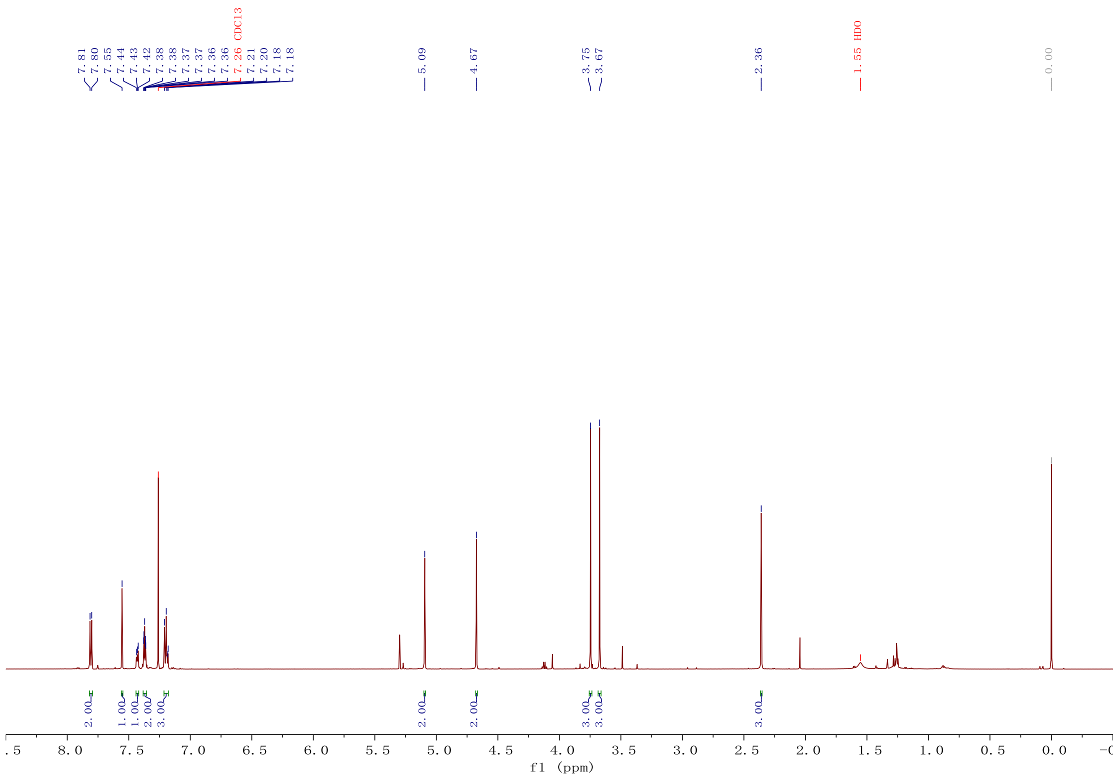

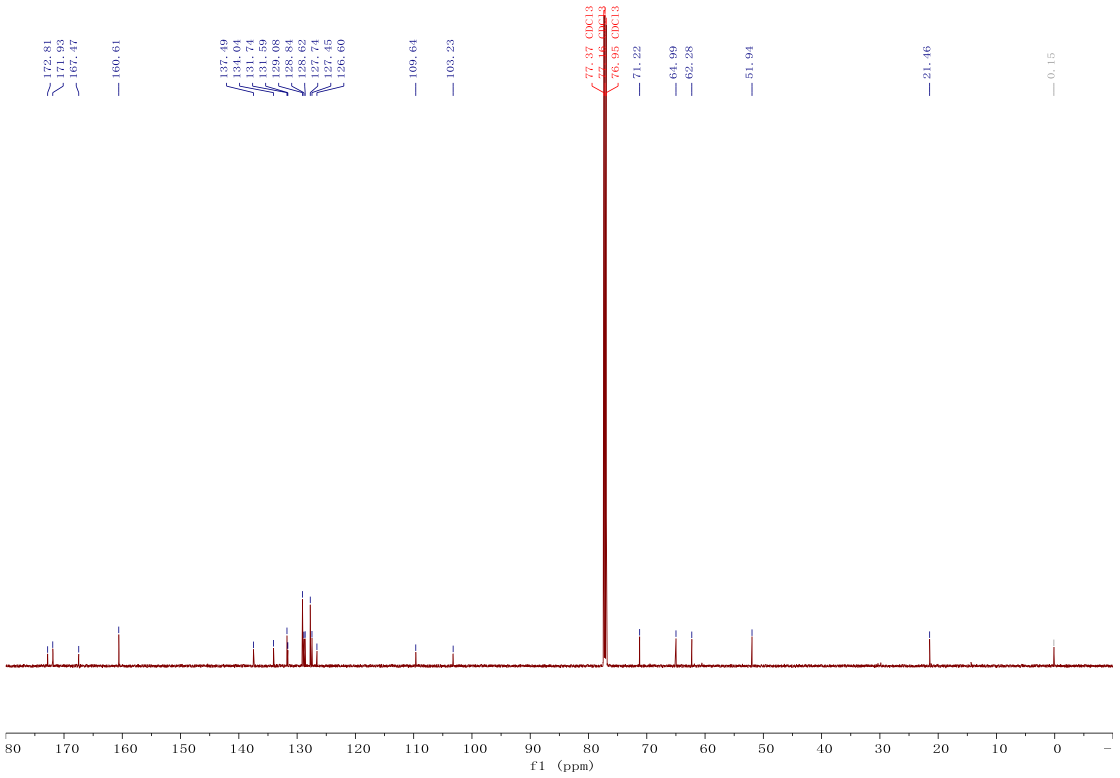

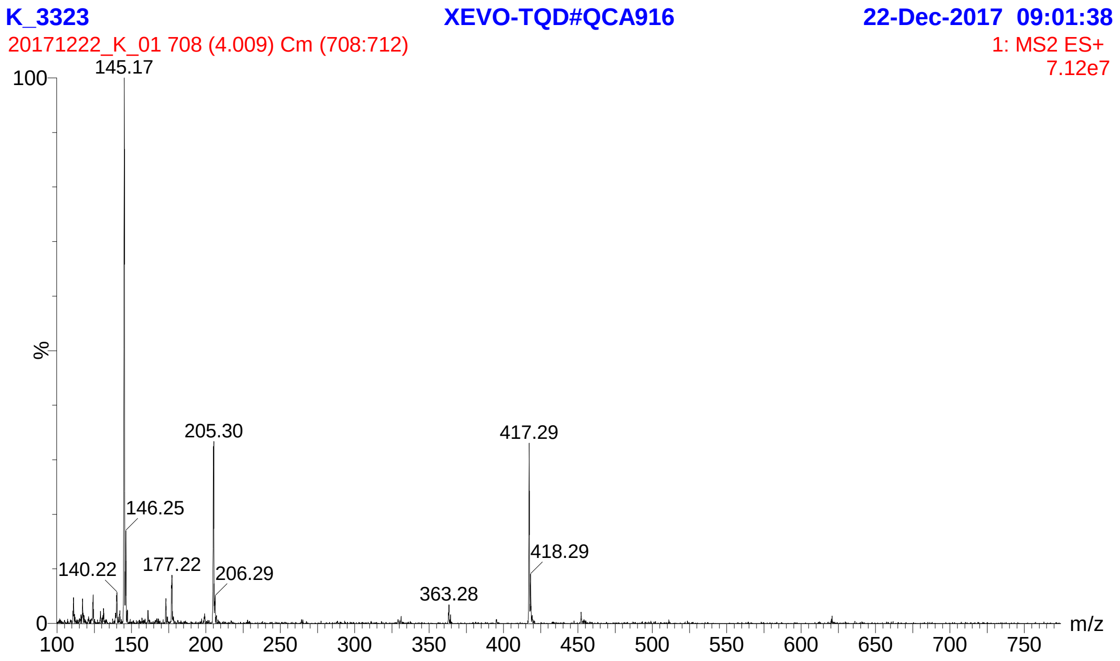

Spectral data for Methyl 2-(2-(((4-(2-fluorophenyl)-5-oxo-2,5-dihydrofuran-3-yl)oxy)methyl)phenyl)-3-methoxyacrylate (7j).

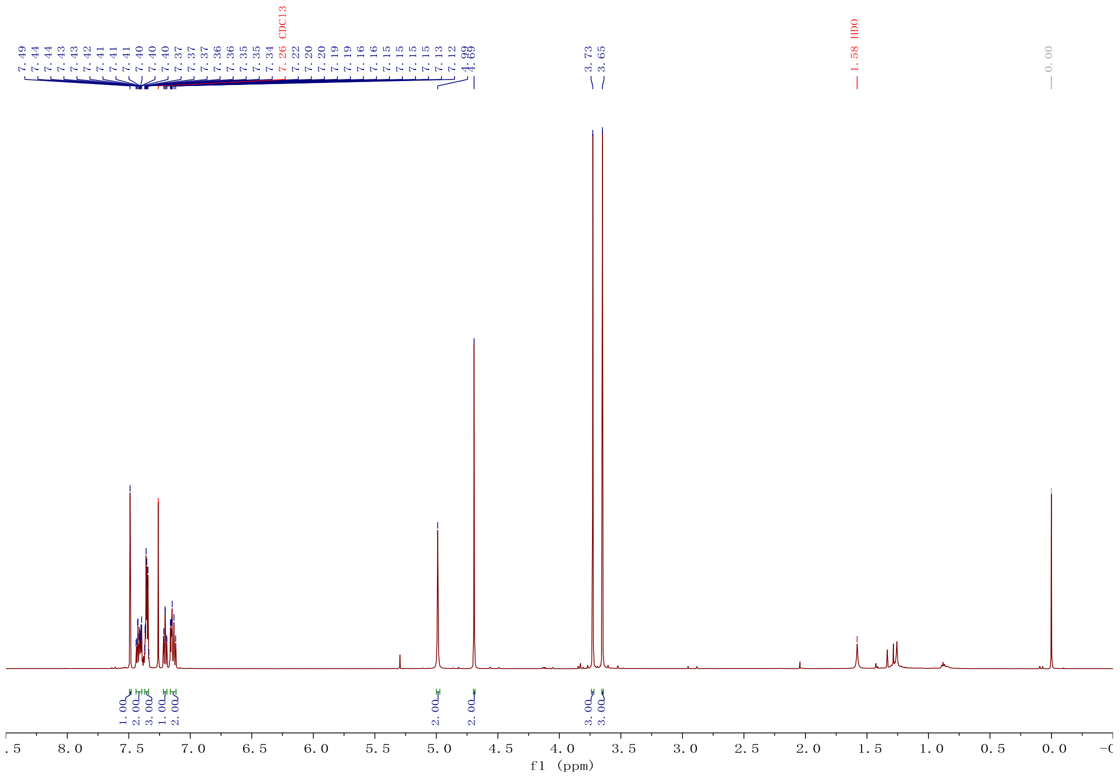

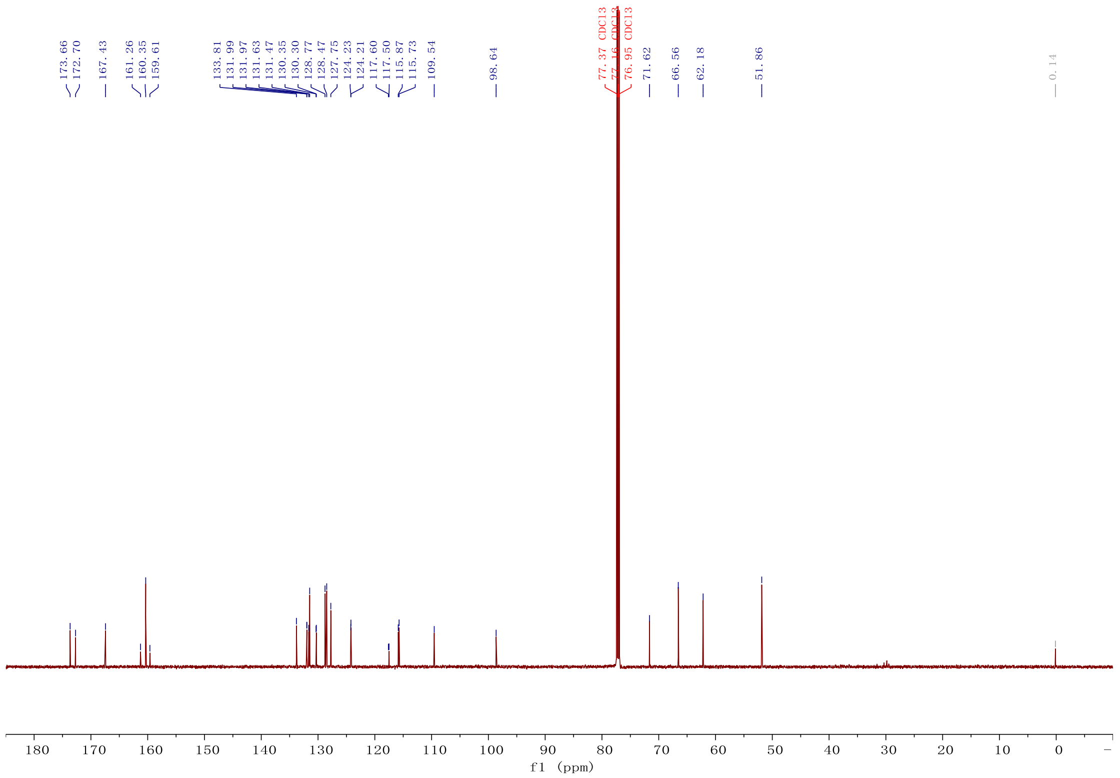

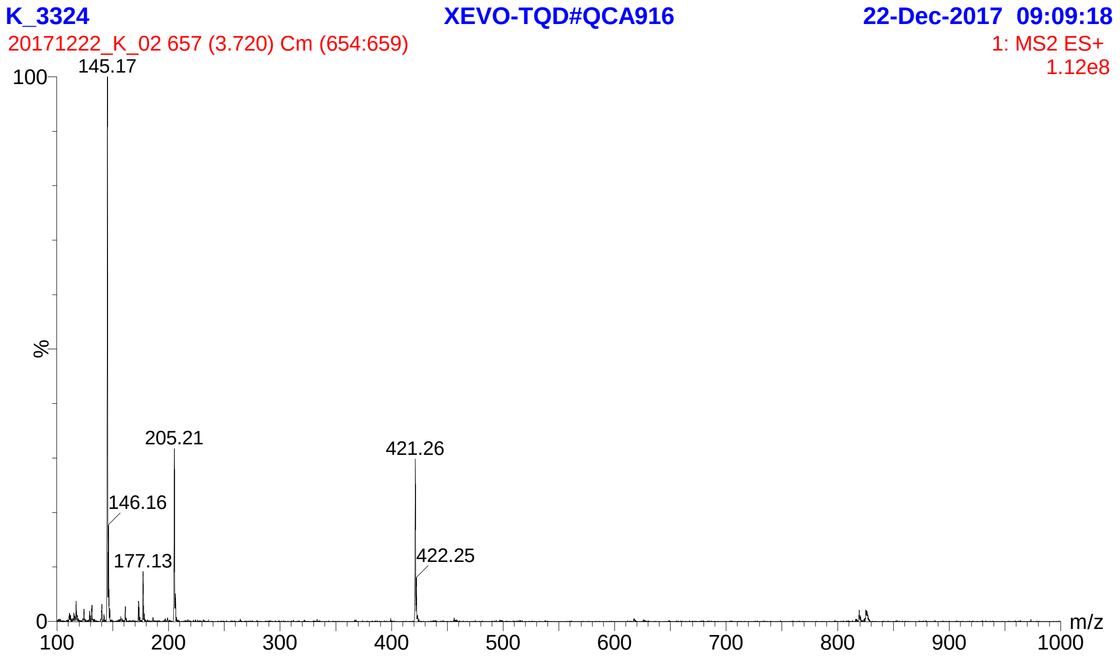

Spectral data for Methyl 3-methoxy-2-(2-(((5-oxo-4-(2,4,5-trifluorophenyl)-2,5-dihydrofuran-3-yl)oxy)methyl)phenyl)acrylate (7k).

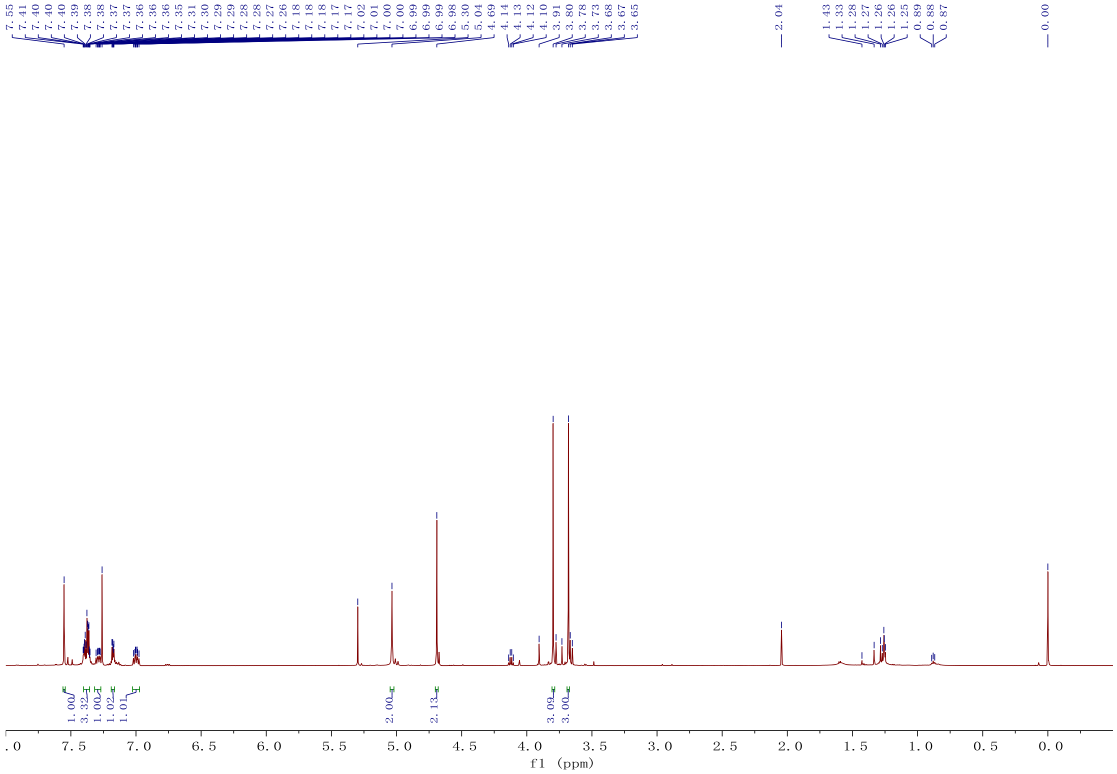

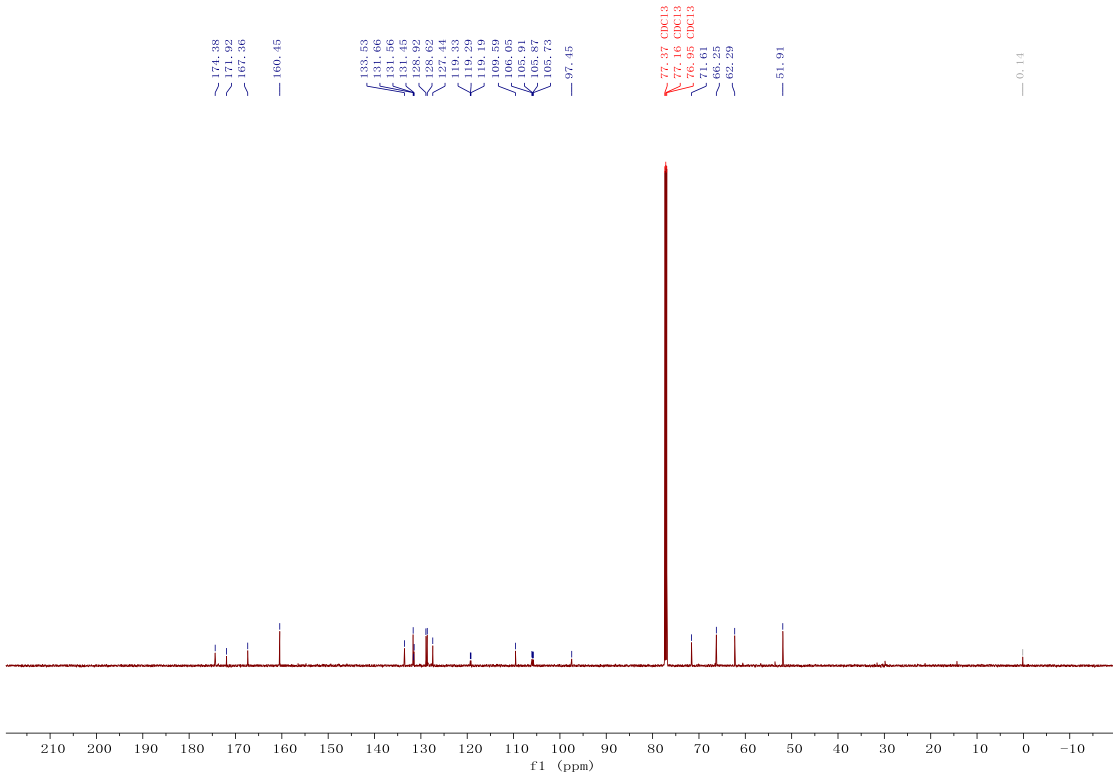

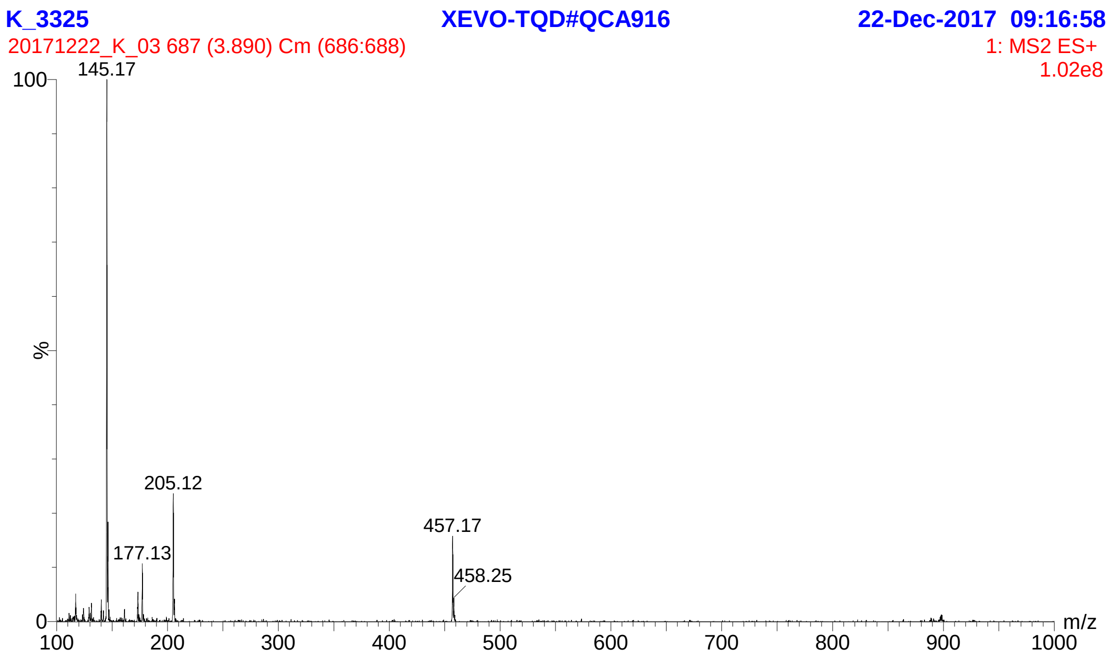
K_3325
XEVO-TQD#QCA916
22-Dec-2017 09:16:58
20171222_K_03 687 (3.890) Cm (686:688)
1: MS2 ES+
1.02e8
145.17
205.12
177.13
457.17
458.25
%
100
0
100
200
300
400
500
600
700
800
900
1000
m/z
